# Association of Perivascular Space Volume in Basal Ganglia Subfields With Cognitive Impairment in Cerebral Small Vessel Disease

**DOI:** 10.1002/hbm.70642

**Published:** 2026-11-09

**Authors:** Lulu Ai, Zhiwei Li, Chaojuan Huang, Xia Zhou, Qiaoqiao Xu, Xiaoqun Zhu, Zhongwu Sun

**Affiliations:** ^1^ Department of Neurology The First Affiliated Hospital of Anhui Medical University Hefei China; ^2^ Department of Neurology The Third Affiliated Hospital of Anhui Medical University (Hefei City First People's Hospital) Hefei China

**Keywords:** basal ganglia, cerebral small vessel disease, cognitive impairment, perivascular space

## Abstract

Perivascular spaces (PVS) serve as essential elements of the glymphatic pathway, facilitating waste clearance and nutrient transport, and their impairment may underlie cognitive deficits in cerebral small vessel disease (CSVD). Here, we investigated associations between PVS characteristics, potential determinants, and cognitive impairment in CSVD. 132 individuals diagnosed with CSVD and 40 healthy controls (HCs) were recruited to participate in a series of thorough neuropsychological assessments and neuroimaging examinations. PVS volume fraction (VF) was quantified in basal ganglia (BG), thalamus, hippocampus, and white matter (WM) using an automated, multimodal image processing pipeline (Enhanced PVS Contrast, EPC) combined with the Frangi filter for robust segmentation. Partial correlation analyses, adjusted for covariates, were performed. CSVD patients exhibited significantly larger PVS VF in the BG and hippocampus, particularly in caudate and putamen subfields of BG (all *p* < 0.05). In CSVD group, advancing age correlated with elevated PVS VF in both BG and hippocampus, especially in males (all *p*_FDR_ < 0.05). A main effect of sex on PVS VF was observed in this patient cohort across the BG, caudate, hippocampus and thalamus, where males exhibited larger PVS VF than females (all *p* < 0.05). In HC group, age correlated positively with PVS volume fraction in the BG (*p*_FDR_ < 0.05), with no significant sex differences across regions. Enlarged BG‐PVS was associated with worse global cognitive function (pr = −0.27, *p*_FDR_ = 0.027) and processing speed (pr = −0.31, *p*_FDR_ = 0.010), persisting after controlling for other neuroimaging markers (including WMHs, lacunes, and CMBs) and regional volume. Subfield analyses revealed the correlation mainly occurring in the putamen (pr = −0.31, *p*_FDR_ = 0.010). Hippocampal PVS VF inversely correlated with hippocampal volume (pr = −0.26, *p*_FDR_ = 0.043), more pronounced in females, and was significantly linked to worse visuospatial function (pr = −0.33, *p*_FDR_ = 0.010). Regional PVS enlargement in CSVD, particularly in BG and hippocampus regions, reflects underlying glymphatic impairment related to age and sex. The prominence of this finding within BG subregions further underscores their role in the pathogenesis of neurocognitive decline.

## Practitioner Points

- An enlarged BG‐PVS, especially caudate and putamen subfields, was found in CSVD.
- PVS volume rises with age, more in males, suggesting sex‐linked glymphatic issues and larger BG‐PVS link to worse cognition, driven by putamen subfield.
- Hippocampal‐PVS growth is inversely related to its volume, impairing visuospatial function.

## Graphical abstract

Schematic overview of the study design and main findings. This cross‐sectional single‐center study demonstrates that enlarged PVS volume in the basal ganglia (BG) subfields, particularly the caudate and putamen, is associated with worse cognitive function in CSVD. PVS burden increases with age and is more prominent in males, while hippocampal PVS enlargement inversely correlates with hippocampal volume and visuospatial function. These findings highlight regional PVS enlargement as a potential biomarker reflecting age and sex‐related glymphatic dysfunction in CSVD.

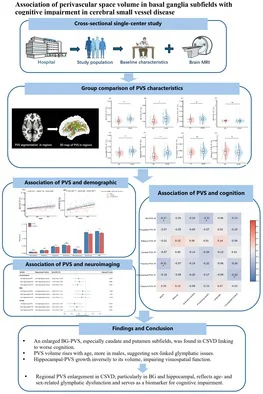

## 1 Introduction

As a leading cause of vascular cognitive impairment, cerebral small vessel disease (CSVD) disrupts brain function through pathological changes in small cerebral vessels (Duering et al. 2023; Wardlaw et al. 2019). Pathological changes associated with CSVD are observable through neuroimaging features like white matter hyperintensities (WMHs), enlarged perivascular spaces (ePVS), lacunes, and cerebral microbleeds (CMBs) (Wardlaw et al. 2019; Ter Telgte et al. 2018). Nevertheless, the fundamental mechanisms driving the onset, progression, and cognitive consequences of CSVD remain largely elusive.

Accumulating evidence suggests that glymphatic dysfunction may contribute to the pathophysiology of CSVD (Xu et al. 2023). As critical components of the glymphatic system, perivascular spaces (PVS) surround penetrating arteries and veins, facilitating fluid exchange and metabolic waste removal while contributing to neurovascular unit integrity and blood–brain barrier maintenance (Benveniste et al. 2019). Direct human evidence for this functional role has recently been provided, demonstrating that PVS serve as conduits for cerebrospinal fluid (CSF) flow in the human brain, confirming that these anatomical structures are not mere epiphenomena but active participants in fluid transport (Yamamoto et al. 2024). When glymphatic clearance is impaired, metabolic waste may accumulate in the brain, potentially promoting the enlargement of PVS (Debette et al. 2019; Zhu, Dufouil, et al. 2010; Zhu, Tzourio, et al. 2010; Ding et al. 2021). Notably, ePVS burden in specific neuroanatomical regions, typically in the white matter (WM) and deep gray matter, such as the basal ganglia (BG), may reflect distinct risk factors and pathophysiological mechanisms, warranting further investigation into region‐specific neurovascular vulnerabilities in CSVD.

Early pathological investigations dismissed ePVS as rare indicators of parenchymal brain injury with limited clinical relevance. However, emerging evidence has implicated ePVS in aging processes and pathological conditions, including CSVD (Doubal et al. 2010; Wardlaw, Smith, Biessels, et al. 2013; Wardlaw, Smith, and Dichgans 2013) and Alzheimer's disease (AD) (Ramirez et al. 2015). This expanded clinical significance is mirrored by growing research into the multifaceted determinants of PVS morphology and distribution. Of particular interest is the frequent co‐occurrence of ePVS with other CSVD imaging markers that collectively correlate with age‐related cognitive deterioration (Hurford et al. 2014). Consequently, numerous investigations have examined the potential link between ePVS and cognitive outcomes, yet the results remain inconsistent. Several studies have reported associations between BG‐ePVS and cognitive decline (Paradise et al. 2021; Zhu, Dufouil, et al. 2010; Zhu, Tzourio, et al. 2010; Passiak et al. 2019), with some suggesting that this relationship is independent of vascular neuropathology and conventional vascular risk factors, pointing to a distinct pathophysiological pathway beyond traditional cerebrovascular injury (Javierre‐Petit et al. 2024). Conversely, other investigations, particularly in cohorts of ischemic stroke patients, have failed to replicate this association (Benjamin et al. 2018; Hurford et al. 2014). Discrepancies also exist regarding hippocampal ePVS, with one study reporting no relationship to baseline cognition or dementia risk (Yao et al. 2014). These inconsistencies may arise from methodological heterogeneity, including regional specificity of PVS distribution and non‐standardized ePVS assessment methods. Most prior studies have focused on select brain regions, predominantly the BG and centrum semiovale (CSO), while excluding other ePVS‐prone areas like the hippocampal. Furthermore, no studies have examined ePVS within BG subfields, where microstructural vulnerabilities may differentially impact cognitive circuits.

Historically, most studies have relied on semi‐quantitative visual rating scales to assess ePVS burden. While clinically practical, these scales are inherently constrained by ceiling effects, limited sensitivity to subtle changes, and considerable inter‐rater variability (Wardlaw, Smith, Biessels, et al. 2013; Wardlaw, Smith, and Dichgans 2013; Potter et al. 2015). More recently, quantitative approaches have been developed to address these shortcomings. Filter‐based methods, such as Frangi filtering, have shown good histopathological validity and enable objective, continuous measurement of PVS burden (Sepehrband et al. 2019; Ballerini et al. 2018). Deep learning‐based methods have also emerged as a promising alternative, offering higher processing speed and the capacity to distinguish PVS from other confounding lesions, but are limited by the need for large, high‐quality manually annotated training datasets and face generalizability challenges across scanners and field strengths (Dubost et al. 2019; Waymont et al. 2024). The wide heterogeneity in quantification methodologies across studies, ranging from visual scales to automated segmentation, and from whole‐brain to region‐specific approaches, has likely contributed to the inconsistencies in reported associations between ePVS and cognitive outcomes. This methodological diversity, coupled with the predominant focus on select brain regions (e.g., the basal ganglia and centrum semiovale) at the expense of other ePVS‐prone areas such as the hippocampus, underscores the need for a standardized, multi‐regional, automated quantification framework.

To address these gaps, the present study applied a fully automated Frangi filter and Enhanced PVS Contrast (EPC)‐based pipeline to quantify ePVS volume across both commonly examined and less‐studied (e.g., hippocampus) regions (Sepehrband et al. 2019). Additionally, we introduced a normalization approach based on normal‐appearing brain tissue (NABT) to mitigate potential confounding from white matter hyperintensities and performed a subfield‐level parcellation of the basal ganglia, providing novel anatomical specificity to investigate region‐specific contributions to vascular cognitive impairment.

## 2 Materials and Methods

### 2.1 Participants

Study inclusion criteria for the 132 CSVD patients were: (1) age 50–80 years; (2) native Mandarin speakers with right‐hand dominance; (3) Magnetic Resonance Imaging (MRI) findings consistent with CSVD diagnostic guidelines (Wardlaw, Smith, Biessels, et al. 2013; Wardlaw, Smith, and Dichgans 2013), with one or more of the imaging features: periventricular WMH (Fazekas score 3) or deep WMH (Fazekas score ≥ 2); one or more CMBs; one or more lacunes. The exclusion criteria were: (1) other major neurological diseases potentially confounding cognitive or neuroimaging evaluations, encompassing but not limited to established neurodegenerative disorders, major stroke (cortical/subcortical infarct causing persistent neurological deficit), multiple sclerosis, epilepsy, intracranial infection, or intracranial hemorrhage; (2) a history of psychiatric conditions not considered secondary to CSVD; (3) a history of neoplastic disease; (4) severe cardiovascular, hepatic, or renal dysfunction; (5) craniocerebral injury; (6) inability to complete the required examinations. Concurrently, 40 healthy controls (HCs) were recruited based on the absence of significant neurological or psychiatric disorders and they underwent the same clinical screening and met the same exclusion criteria as the CSVD patients (as detailed above), with the additional requirement of not meeting the neuroimaging inclusion criteria for CSVD.

### 2.2 Baseline Clinical Characteristics

A trained neurologist documented demographic information for each participant, including age, sex, and years of education. Vascular risk factor (VRFs) data, namely hypertension, diabetes mellitus (DM), hypercholesterolemia, smoking, and body mass index (BMI), were collected via a standardized questionnaire (Gottesman et al. 2017; van Norden et al. 2011) and defined following current international guidelines. Hypertension was characterized by systolic BP ≥ 140 mmHg, diastolic BP ≥ 90 mmHg, or antihypertensive medication use (Williams et al. 2018). Diabetes mellitus (DM) was characterized by fasting glucose ≥ 7.0 mmol/L, HbA1c ≥ 6.5%, or ongoing treatment with glucose‐regulating pharmacotherapy (ElSayed et al. 2023). Hypercholesterolemia was characterized by total cholesterol > 5.2 mmol/L, LDL‐C > 2.58 mmol/L, or ongoing treatment with lipid‐lowering pharmacotherapy (Grundy et al. 2019). All vascular risk variables were dichotomized as present or absent for analysis.

### 2.3 Cognitive Function Assessment

Within 1 week of undergoing MRI, all participants completed a neuropsychological evaluation administered by a trained neurologist in a quiet environment. The Chinese adaptation of the Montreal Cognitive Assessment (MoCA) was administered to evaluate global cognition (Huang et al. 2018). Domain‐specific cognitive function was evaluated using a test battery: memory was assessed using the Auditory Verbal Learning Test (AVLT) (Putcha et al. 2019); executive function and processing speed were assessed using the Stroop Color Word Test, specifically, the color‐word condition (SCWT‐C) for executive function and the dot‐condition (SCWT‐A) for processing speed (Scarpina and Tagini 2017); visuospatial function was measured with the 10‐point Clock Drawing Test (CDT‐10) (Hshieh et al. 2018); language performance was assessed using the Boston Naming Test (BNT) (Williams et al. 1989). To enable cross‐group comparisons of cognitive performance, *z*‐transformations were applied to each cognitive domain score, using the formula: (individual raw score—control group mean)/control group standard deviation (Wang et al. 2023). Because raw scores on the SCWT reflect completion time where longer durations denote poorer performance, the *z*‐scores for SCWT‐A and SCWT‐C were subsequently inverted through multiplication by −1. This adjustment ensured that higher *z*‐scores uniformly represent superior cognitive performance across all assessed domains (Huang et al. 2020).

### 2.4 MRI Acquisition

MRI imaging was performed on a 3.0 T GE Discovery MR750w scanner with a 24‐channel head coil (Supporting Information eMethod for details).

### 2.5 Volumetric Quantification of Brain Structures

Automatic segmentation and volumetric quantification of the whole brain were performed on high‐resolution T1 images utilizing the FreeSurfer software package using the fully automated recon‐all processing stream (version 7.2.0, http://surfer.nmr.mgh.harvard.edu/). The processing stream begins with unified preprocessing of T1‐weighted images, encompassing intensity normalization, skull stripping, and nonlinear registration to Talairach standard space (Nishizawa et al. 2022). Volumes of the thalamus and BG were extracted using the software's built‐in voxel‐based automated neuroanatomical labeling stream. Hippocampal segmentation was conducted using an automated module based on a computational atlas derived from ultra‐high‐resolution ex vivo MRI data (~0.1 mm isotropic) (Iglesias et al. 2015). All image segmentations were visually inspected for quality assurance. Normalization to the estimated total intracranial volume (TIV) was applied to all regional volumes to control for head size differences. The formula for hippocampal volume is as follows:

$$\text{Hippocampal normalized volume}=\frac{\text{Hippocampal volume}}{\mathrm{TIV}}\times 1000$$

### 2.6 Quantification of PVS Volume Fraction

A fully automated quantitative pipeline, implementing the Enhanced PVS Contrast (EPC) method (Sepehrband et al. 2019), was employed for PVS mapping (Figure 1). By integrating T1‐ and T2‐weighted images and employing a dedicated filter to remove unstructured high‐frequency noise, this technique improves the visibility of PVS on MRI. After intensity normalization and skull stripping, FLAIR images were then inhomogeneity‐corrected with Advanced Normalization Tools (ANTs) and linearly/nonlinearly registered to Montreal Neurological Institute (MNI)‐152 space using FSL's FNIRT. Subsequently, we used FSL's FAST and FIRST toolboxes for automated tissue and subcortical structure segmentation to extract the masks of the regions of interest (ROIs). Specifically, basal ganglia (BG) and subfields (including the caudate, putamen, pallidum, and amygdala), as well as the thalamus and hippocampus, were segmented using FSL's FIRST, a model‐based tool that utilizes shape and appearance models constructed from manually segmented training data to provide robust and accurate segmentation of subcortical structures (Patenaude et al. 2011). The white matter (WM) mask was generated using FSL's FAST for tissue‐type segmentation (Zhang et al. 2001). Following adaptive non‐local mean filtering of T1‐weighted and T2‐weighted images, EPC maps were derived from the filtered image ratio (T1w/T2w). Subsequently, the Frangi filter was applied to regions of interest with default parameter settings to generate quantitative vesselness maps for perivascular space assessment. Finally, the volumes of PVS were automatically obtained through summation of delineated PVS volume for each region of interest (Benjamin et al. 2018). To address the potential confounding effect of gliotic lesions on PVS quantification, we calculated a modified PVS VF by normalizing PVS volume to the volume of normal‐appearing brain tissue (NABT), defined as parenchymal volume minus WMH volume. WMH volume was quantified automatically from T2‐FLAIR images using the Lesion Segmentation Algorithm (LPA) implemented in the Lesion Segmentation Toolbox (LST) for SPM, followed by manual correction to ensure accuracy. PVS volume fraction was defined mathematically as:

$$\begin{array}{c} \mathrm{PVS}\;\text{volume fraction}\;\left(\mathrm{PVS}\;\mathrm{VF}\right)\\ =\frac{\;\mathrm{PVS}\;\text{volume}}{\text{grey}+\text{white matter volume}-\mathrm{WMH}\;\text{volume}}\times 100\% \end{array}$$

**FIGURE 1:**
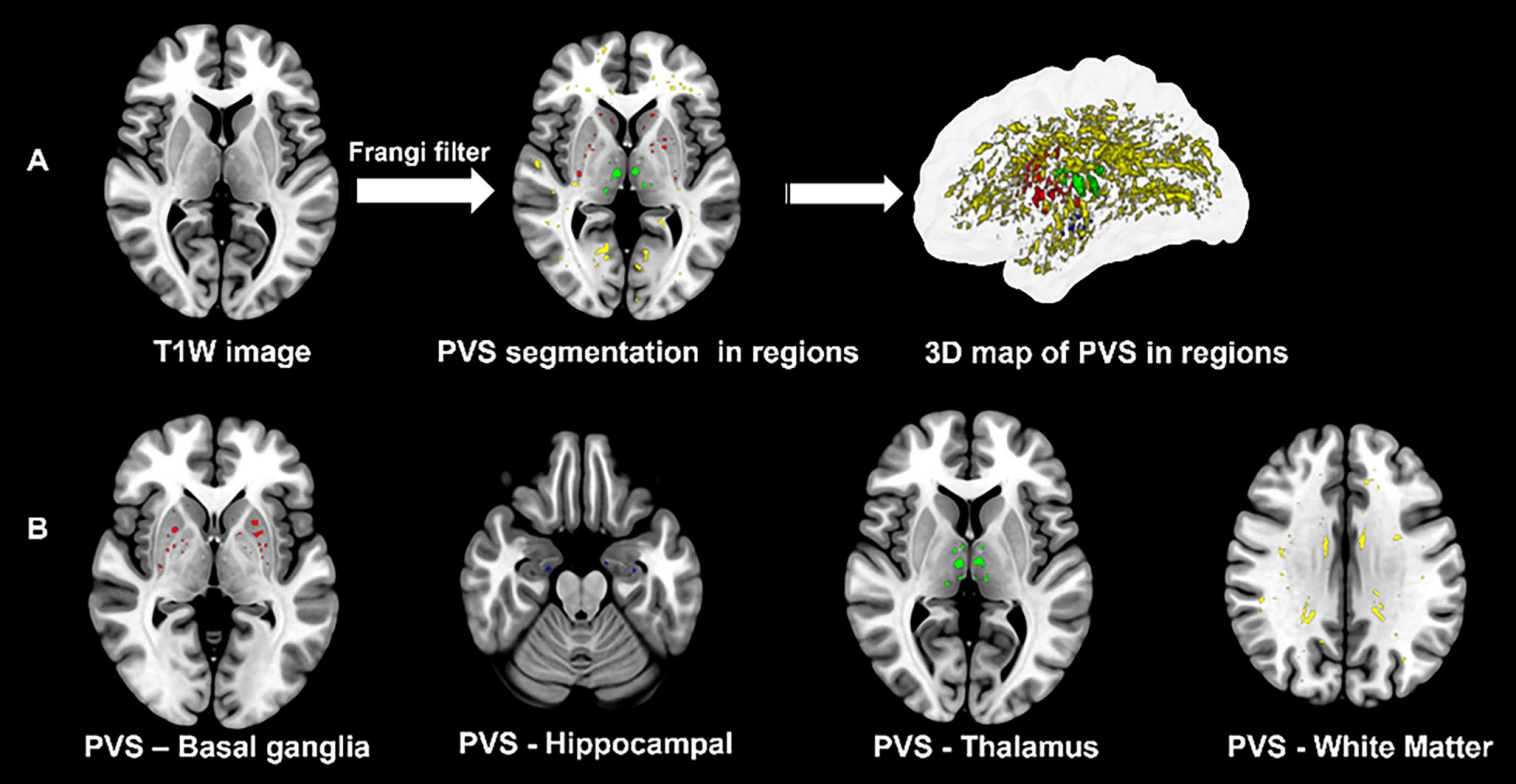
(A) Schematic diagram illustrating the automated PVS segmentation methodology. The process involves co‐registration of T1w and T2w images, generation of the Enhanced PVS Contrast (EPC) map, and application of the Frangi filter for automated PVS delineation within segmented brain regions. (B) Resultant 3D distribution of automatically segmented PVS in key regions: Basal ganglia (red), Hippocampal (light blue), Thalamus (light green), and White matter (yellow). EPC, Enhanced PVS Contrast; PVS, perivascular spaces.

### 2.7 Assessment of CSVD Neuroimaging Markers

To account for the overall burden of CSVD in our statistical models, we assessed and quantified the following established CSVD markers, in accordance with the STRIVE standards (Wardlaw, Smith, Biessels, et al. 2013; Wardlaw, Smith, and Dichgans 2013). On T2‐FLAIR sequences, WMHs appear as hyperintense areas within white matter regions, presenting as punctate lesions or confluent irregular patches (Duering et al. 2023). WMHs severity was graded using the Fazekas visual rating scale, with periventricular WMHs (PWMHs) scored 2–3 or deep WMHs (DWMHs) scored 3. CMBs appear as round or ovoid hypointense lesions (2–10 mm) with clear boundaries and no surrounding edema on SWI (Duering et al. 2023; Greenberg and Charidimou 2018). Lacunes appear as round or ovoid cavities (3–15 mm), featuring central CSF‐equivalent hypointensity and a hyperintense peripheral rim (Wardlaw, Smith, Biessels, et al. 2013; Wardlaw, Smith, and Dichgans 2013).

### 2.8 Statistical Analysis

Normality of continuous variables was examined using the Shapiro–Wilk test. Variables satisfying normality criteria (*p* > 0.05) were summarized as mean ± standard deviation and compared with two‐sample two‐tailed *t*‐tests. Variables deviating from normality were described using median and interquartile range (IQR), with group comparisons performed via the Kruskal–Wallis test. Categorical variables were described as frequencies (*n*, %) and compared using Pearson's chi‐square tests. PVS volume comparisons between groups were age‐ and sex‐adjusted. To comprehensively assess the determinants of PVS burden, we first examined the associations between PVS volume fraction and all candidate vascular risk factors, including age, sex, BMI, hypertension, diabetes, hypercholesterolemia, and smoking. Subsequently, we constructed multivariable‐adjusted models incorporating all these factors as covariates. For analyses involving neuroimaging markers, partial correlations were performed controlling for demographics (age, sex, education) and the aforementioned vascular risk factors. For cognitive performance analyses, the models were further adjusted for other CSVD imaging markers (WMHs, lacunes, and CMBs) and regional brain volumes, in addition to the demographic and vascular covariates. Correlation analyses underwent false discovery rate (FDR) correction for multiple comparisons, with FDR‐adjusted *p*‐values (*p*_FDR_) reported for all primary association analyses. For group comparisons of baseline characteristics, nominal *p*‐values (uncorrected) are reported, consistent with standard practice in descriptive analyses. All statistical procedures were performed in R (version 4.1.1) and IBM SPSS Statistics (version 26), with significance at *p* < 0.05.

## 3 Results

### 3.1 Participant Characteristics

As shown in Table 1, a total of 132 patients with CSVD and 40 HCs were included. Compared to HCs, CSVD patients were significantly older (*p* < 0.001) and demonstrated a higher prevalence of smoking (*p* = 0.008) and hypertension (*p* = 0.001). No group differences emerged for sex distribution, years of education, diabetes, hypercholesterolemia, or BMI (all *p* > 0.05). In the CSVD group, 79.07%, 76.19%, and 65.62% were receiving treatment for hypertension, diabetes, and hypercholesterolemia, respectively. The control rates of blood pressure, blood glucose, and LDL‐C among the treated patients were 64.71%, 62.50%, and 33.33%, respectively. No significant differences in these rates were observed compared to the HC group. Relative to HCs, individuals with CSVD demonstrated poorer cognition across all assessed domains (all *p* < 0.05). CSVD patients also showed increased WMH, lacune, and CMB burdens, along with reduced hippocampal volume (all *p* < 0.05).

**TABLE 1:** Baseline demographic, cognitive, and neuroimaging characteristics of HC and CSVD groups.

	HC (*n* = 40)	CSVD (*n* = 132)	*T*/*λ*‐value	*p*
Demographics				
Age, years	60.18 ± 4.57	63.86 ± 6.76	−3.960	< 0.001
Male, *n* (%)	19 (47.50)	70 (53.03)	0.376	0.540
Education, years	9.45 ± 4.13	8.47 ± 3.83	1.392	0.166
Hypertension, *n* (%)	14 (35.00)	86 (65.20)	11.467	0.001
Use of antihypertensive medication, *n* (%)	12 (85.71)	68 (79.07)	1.582	0.730
Controlled (on medication), *n* (%)	8 (66.67)	44 (64.71)	1.090	1.000
Diabetes mellitus, *n* (%)	6 (15.00)	21 (15.90)	0.019	0.890
Use of antidiabetic medication, *n* (%)	5 (83.33)	16 (76.19)	1.539	1.000
Controlled (on medication), *n* (%)	4 (80.00)	10 (62.50)	2.310	0.624
Hypercholesterolemia, *n* (%)	10 (25.00)	32 (24.24)	0.005	0.941
Use of lipid‐ lowering medication, *n* (%)	6 (60.00)	21 (65.62)	0.790	1.000
Controlled (on medication), *n* (%)	4 (66.67)	7 (33.33)	3.785	0.187
BMI (kg/m^2^)	23.77 ± 2.28	23.97 ± 2.79	−0.416	0.678
Smoking, *n* (%)	5 (12.50)	45 (34.09)	6.940	0.008
Neuropsychological assessments				
MoCA total scores	25.98 ± 1.86	22.04 ± 4.31	8.264	< 0.001
Memory function	0.00 ± 1.00	−0.80 ± 1.46	3.951	< 0.001
Executive function	0.00 ± 1.00	−0.86 ± 1.88	3.802	< 0.001
Processing speed	0.00 ± 1.00	−0.78 ± 1.95	3.359	0.001
Language function	0.00 ± 1.00	−0.54 ± 1.32	2.391	0.018
Visuospatial function	0.00 ± 1.00	−2.65 ± 4.12	6.759	< 0.001
Neuroimaging markers				
WMH Fazekas scores	1.00 (0.00, 2.00)	4.00 (3.00, 6.00)	< 0.001	< 0.001
DWMH scores	1.00 (0.00, 2.00)	4.00 (3.00, 6.00)	< 0.001	< 0.001
PWMH scores	1.00 (0.00, 2.00)	4.00 (3.00, 6.00)	< 0.001	< 0.001
Lacunes, *n* (%)	0 (0.00)	33 (25.00)	12.374	< 0.001
CMBs, *n* (%)	0 (0.00)	63 (47.73)	30.125	< 0.001
Total HP volume	5.00 ± 0.49	4.78 ± 0.51	2.243	0.026

*Note:* Results are expressed as means ± standard deviation for the continuous variables and as frequency (percentage) for the categorical variables. Each cognitive domain is represented as composite *Z* scores. Hypertension control: Blood pressure < 140/90 mmHg while on antihypertensive medication therapy. Diabetes control: Fasting blood glucose < 7.0 mmol/L while on antidiabetic medication therapy. Hypercholesterolemia control: LDL‐C < 2.58 mmol/L while on lipid‐lowering medication therapy. *p*‐Values for group comparisons are unadjusted, as these analyses were descriptive in nature.

Abbreviations: BMI, body mass index; CMBs, cerebral microbleeds; CSVD, cerebral small vessel disease; DWMH, deep white matter hyperintensity; HC, healthy control; HP, hippocampal; MoCA, Montreal Cognitive Assessment; PWMH, periventricular white matter hyperintensity; WMH, white matter hyperintensity.

### 3.2 Group Comparisons in PVS Characteristics

After age and sex adjustment, significant regional enlargement of PVS was observed within the CSVD group relative to HCs, specifically within the BG (*p* < 0.001) and hippocampus (*p* = 0.019), with no statistical differences in thalamus or white matter (Figure 2). Within the BG subfields, the caudate and putamen showed pronounced PVS expansion (all *p* < 0.001), while the amygdala and pallidum showed no intergroup difference (Table S1).

**FIGURE 2:**
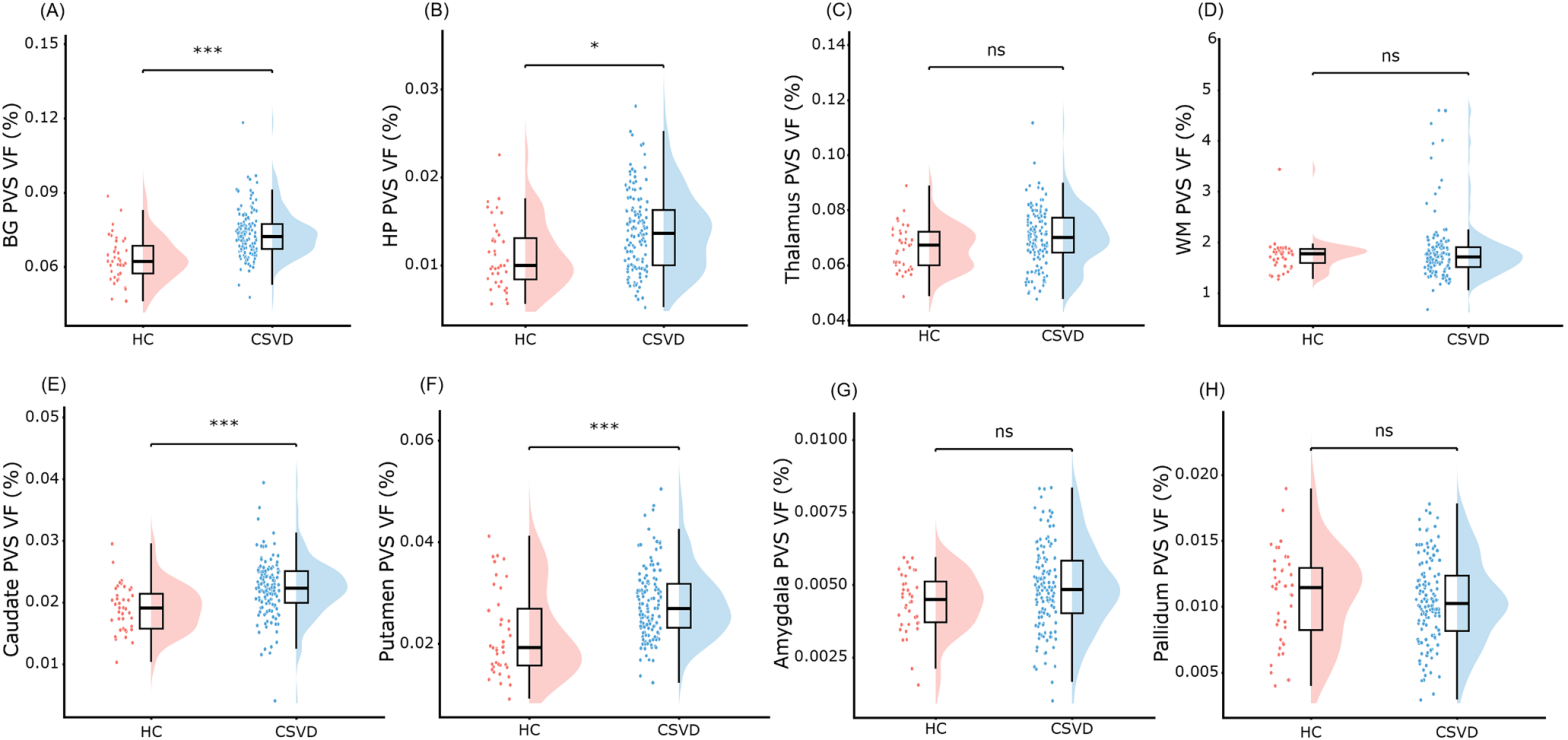
Rain‐cloud plots illustrating group differences in regional PVS volume between HC and CSVD participants. Group comparisons were performed adjusting for age and sex. *p* values shown are unadjusted for multiple comparisons, given the exploratory nature of this analysis. (A‐D) Group comparisons of the total PVS volume fraction in BG, hippocampus, thalamus, and WM. (E‐H) Group comparisons of the total PVS volume fraction in BG subfields, specifically the caudate, putamen, amygdala, and pallidum. Abbreviations: HC, healthy control; HP, hippocampus; CSVD, cerebral small vessel disease; PVS, perivascular space; VF, volume fraction; BG, basal ganglia; WM, white matter. ns not statistically significant, *p < 0.05, ***p < 0.001.

### 3.3 Associations Between PVS Characteristics and Clinical Characteristics in CSVD Group

In the CSVD group, age correlated positively with PVS volume in both BG (pr = 0.24, *p*_FDR_ = 0.044) and hippocampus (pr = 0.33, *p*_FDR_ = 0.002), especially pronounced in males (Figure 3). After age and VRFs (hypertension, diabetes, hypercholesterolemia, smoking, BMI) adjustment, sex exerted a significant main effect on PVS VF in the BG, hippocampus, and thalamus of CSVD patients. Across all three regions, male participants demonstrated significantly greater PVS volume fractions compared to females (all *p* < 0.05). No significant sex‐by‐vascular risk factors interactions were detected for any region (all *p*_FDR_ > 0.05) (Table S3). This sex effect was also evident in the caudate within BG subfield (*p* < 0.05) (Figure 4). While BMI correlated positively with PVS volume in BG and white matter, it did not survive multiple comparison correction (Table S2). The complete set of correlation results between PVS burden and all examined clinical characteristics is provided in Table S2.

**FIGURE 3:**
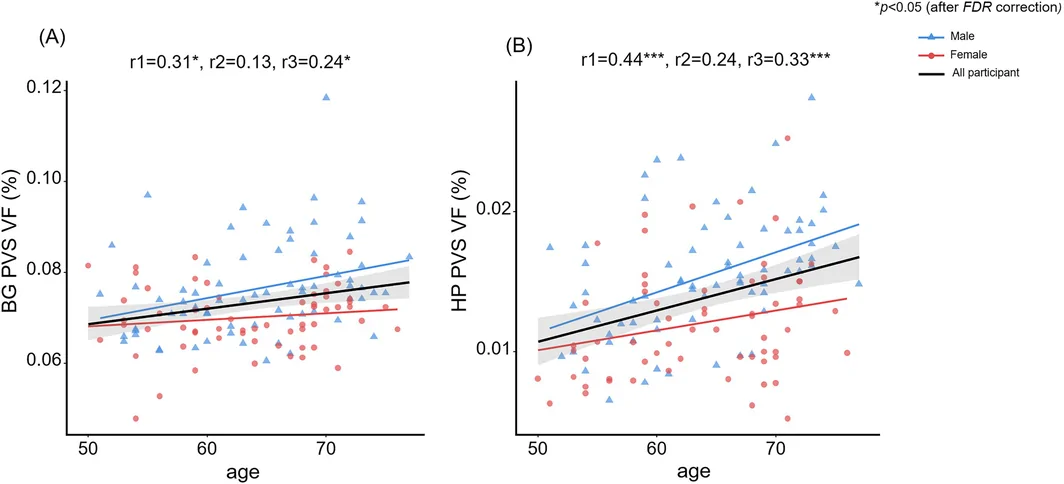
Associations of regional PVS VF with age among CSVD patients. (A) Partial correlation analysis indicates positive correlations between BG PVS VF and age in males (*r*_1_ = 0.31) and all participants (*r*_3_ = 0.24), whereas no correlation in females (*r*_2_ = 0.13). (B) Partial correlation analysis indicates positive correlations between hippocampal PVS VF and age in males (*r*_1_ = 0.44) and all participants (*r*_3_ = 0.33), whereas no correlation in females (*r*_2_ = 0.24). Abbreviations: BG, basal ganglia; PVS, perivascular space; VF, volume fraction; HP, hippocampus; CSVD, cerebral small vessel disease. *p* values shown are FDR‐corrected for multiple comparisons. *p < 0.05, ***p < 0.001 after FDR correction.

**FIGURE 4:**
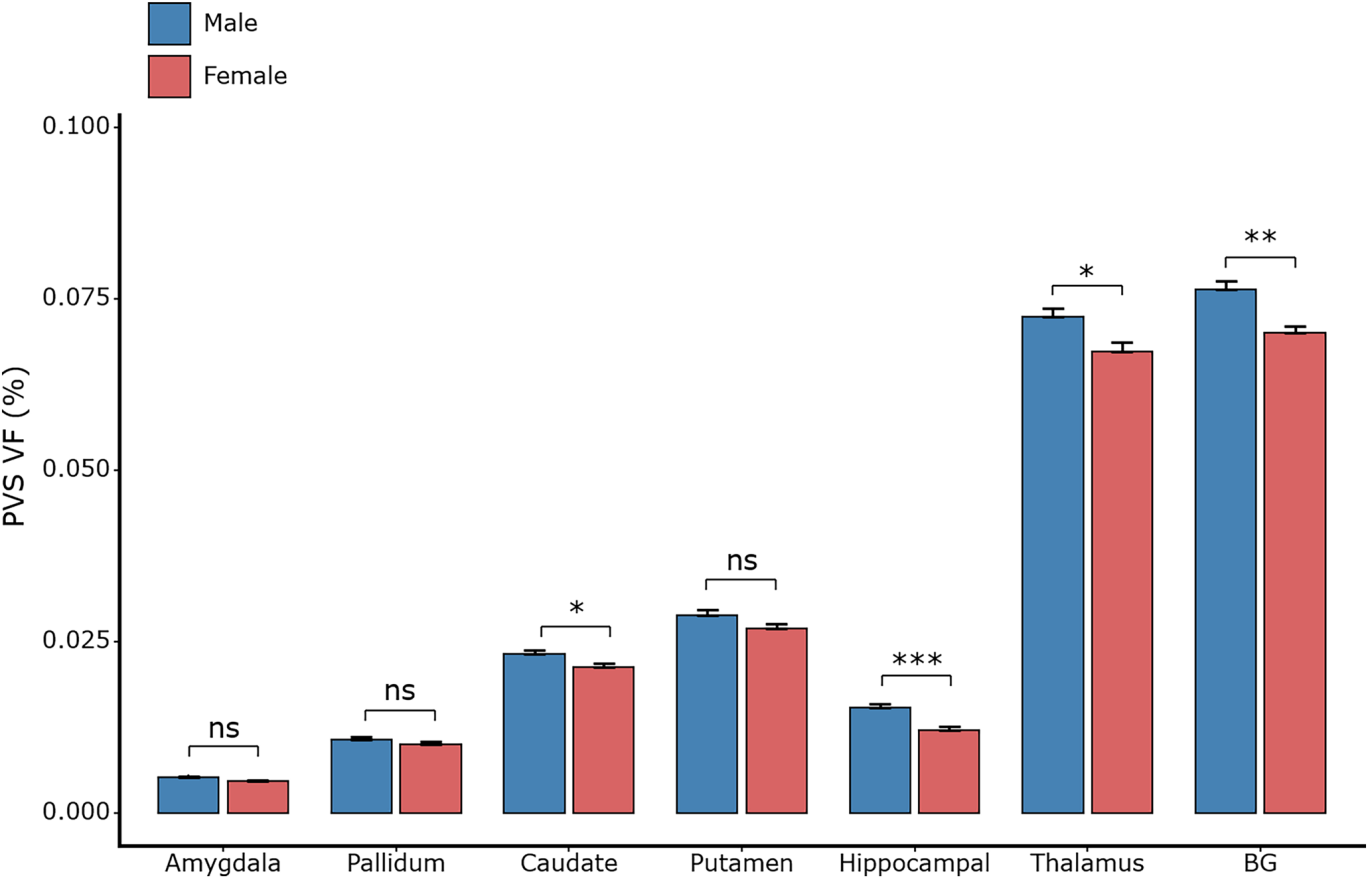
Sex distribution difference of PVS VF across brain regions in CSVD group, adjusted for age and vascular risk factors (hypertension, diabetes, hypercholesterolemia, smoking, BMI). *p*‐Values shown are unadjusted for multiple comparisons. BG, basal ganglia; CSVD, cerebral small vessel disease; PVS, perivascular space; VF, volume fraction. ns, not statistically significant, *p < 0.05, **p < 0.01, ***p < 0.001.

In the HC group, age correlated positively with PVS volume fraction in the BG (pr = 0.47, *p* = 0.002) and hippocampus (pr = 0.40, *p* = 0.010), with the basal ganglia association remaining significant after FDR correction. No significant sex differences were observed in any brain region (Table S4).

### 3.4 Associations Between PVS Characteristics and Neuroimaging Markers in CSVD Group

After age, sex, and VRFs (hypertension, diabetes, hypercholesterolemia, smoking, BMI) adjustment, hippocampal PVS VF correlated inversely with hippocampal volume in CSVD patients (pr = −0.26, *p*_FDR_ = 0.043), surviving multiple comparison correction (Table S2). Meanwhile, patients with CSVD exhibited reduced bilateral hippocampal volume compared to the HCs (Figure 5D–F), while there was a significant increase in PVS VF in left and total hippocampus (Figure 5A–C). Further sex‐stratified analysis revealed that this association between hippocampal PVS VF and hippocampal volume was female‐specific, with no effect in males (Figure 6).

**FIGURE 5:**
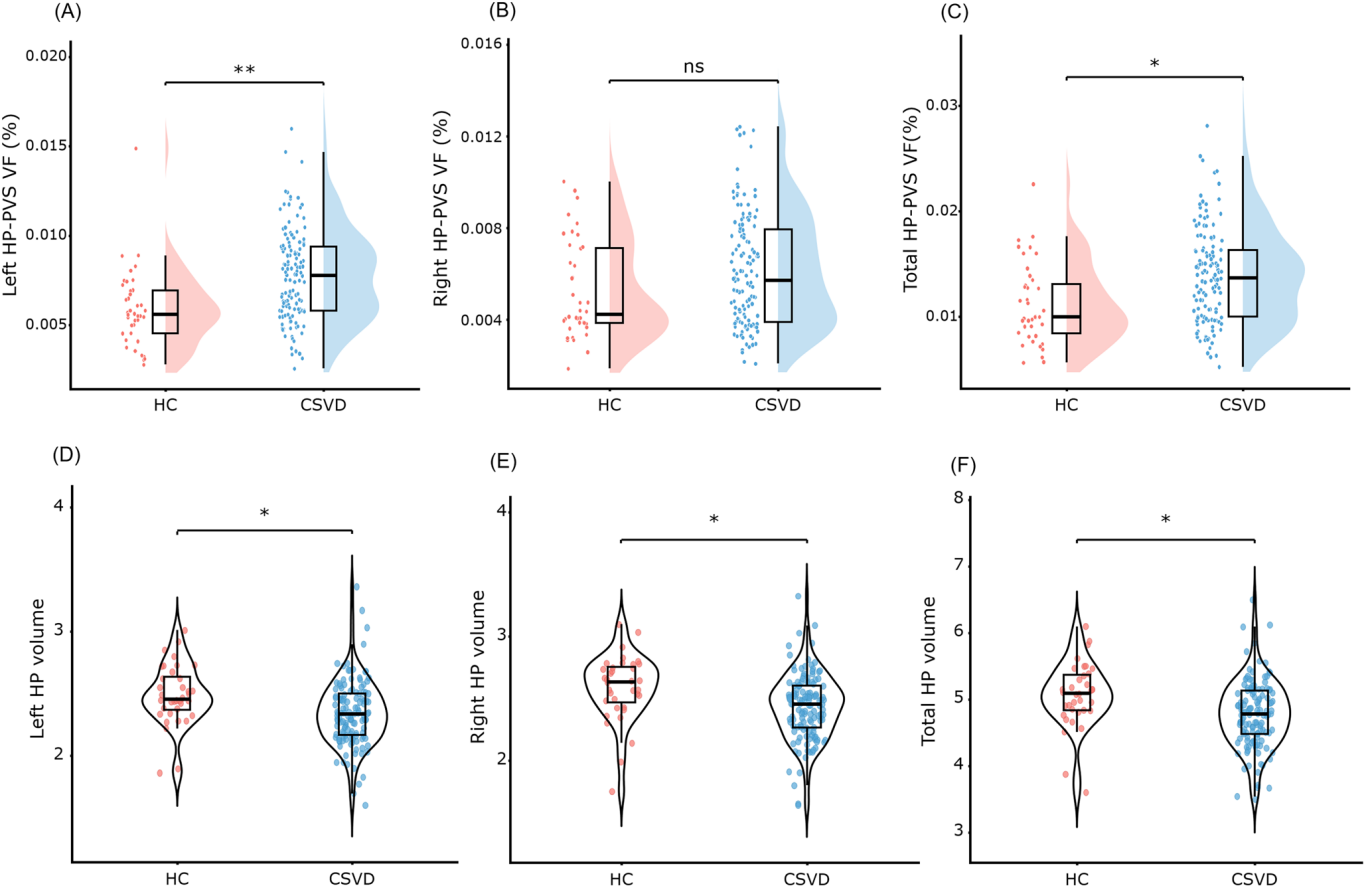
Comparison of bilateral hippocampal PVS VF and hippocampal volume between HC and CSVD groups, adjusting for age and sex. *p* values shown are unadjusted for multiple comparisons. (A‐C) Group‐comparison of bilateral hippocampal PVS VF. (D‐F) Group‐comparison of bilateral hippocampal volume. Abbreviations: HC, healthy control; CSVD, cerebral small vessel disease; HP, hippocampal; PVS, perivascular space; VF, volume fraction. ns, not statistically significant, *p < 0.05, **p < 0.01.

**FIGURE 6:**
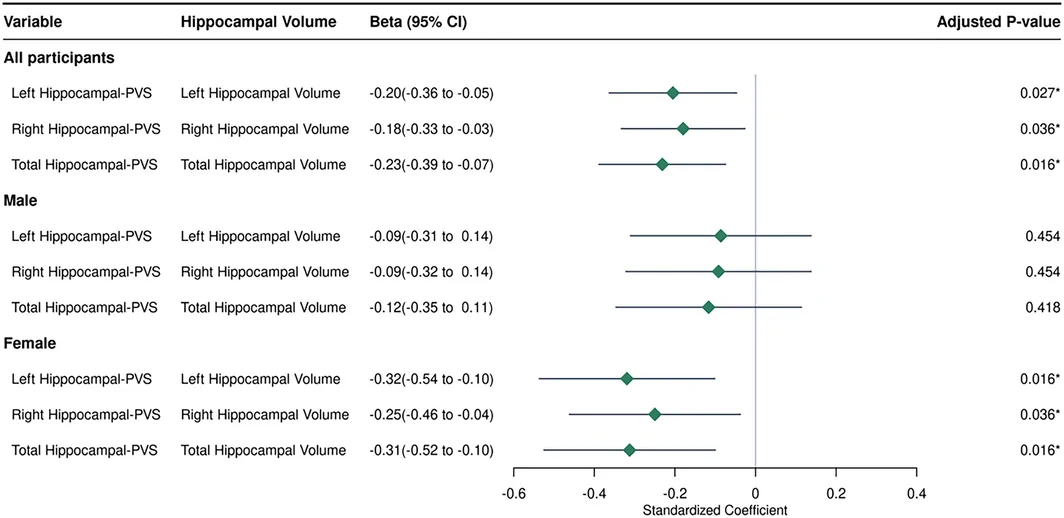
Forest plots of the association between hippocampal PVS VF and hippocampal volume stratified by sex in CSVD group. *β* coefficient with corresponding 95% confidence intervals (95% CI) for the association between hippocampal PVS volume fraction and hippocampal volume are shown, adjusted for age, sex, and vascular risk factors (hypertension, diabetes, hypercholesterolemia, smoking, BMI). Abbreviations: CSVD, cerebral small vessel disease; PVS, perivascular spaces; VF, volume fraction. *p* values shown are FDR‐corrected for multiple comparisons. *p < 0.05 after FDR correction.

### 3.5 Associations Between PVS Characteristics and Cognitive Performance in CSVD Group

After adjustment for demographics (age, sex, education), vascular risk factors (hypertension, diabetes, hypercholesterolemia, smoking, BMI), other CSVD markers (WMHs, lacunes, and CMBs), and volumes of the respective region, partial correlations were visualized as heatmaps in Figure 7. Within the CSVD group, BG‐PVS volume fraction demonstrated a negative correlation with global cognitive function (pr = −0.27, *p*_FDR_ = 0.027) and processing speed (pr = −0.31, *p*_FDR_ = 0.010). Subfield analyses showed putamen PVS linked to global cognitive function (pr = −0.31, *p*_FDR_ = 0.010), while no correlation was found in caudate, amygdala or pallidum. Hippocampal PVS VF correlated inversely with visuospatial performance (pr = −0.33, *p*_FDR_ = 0.010) (Table S5).

**FIGURE 7:**
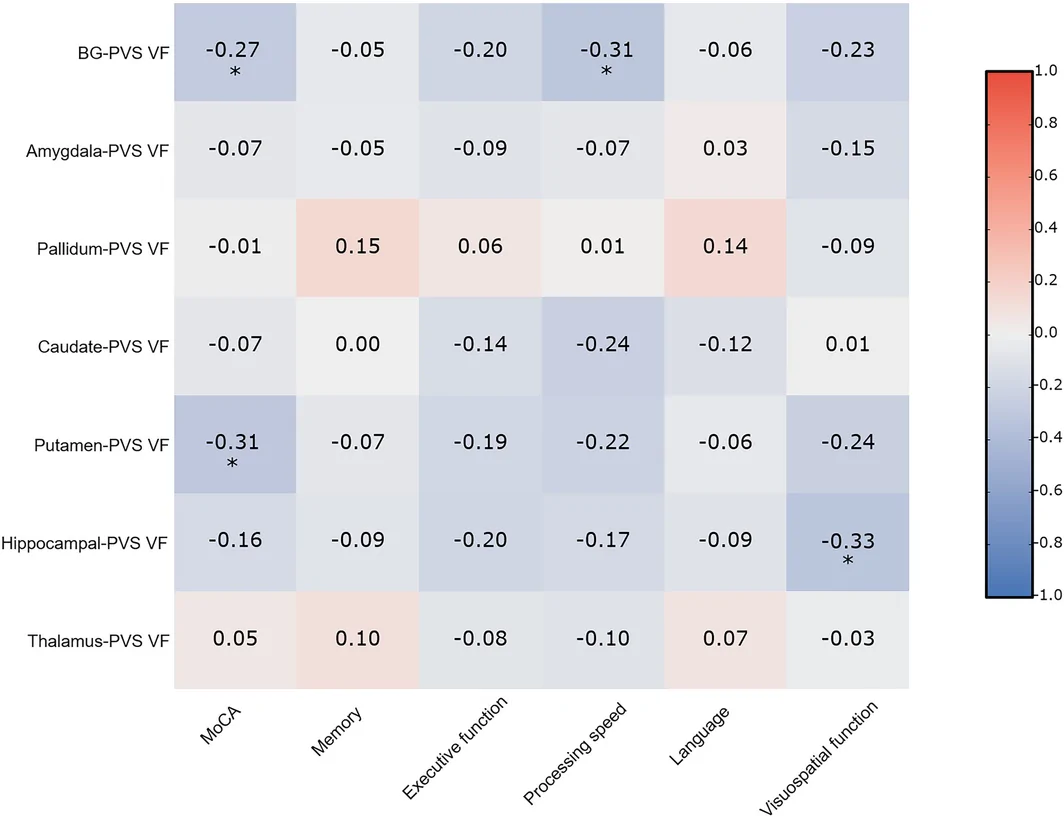
Heatmap visualization of associations between PVS volume fraction across brain regions and cognitive performance in CSVD group, corrected for demographics (age, sex, and years of education), vascular risk factors (hypertension, diabetes, hypercholesterolemia, smoking, BMI), other CSVD neuroimaging markers (WMHs, lacunes, and CMBs), and respective brain region volumes. *p*‐Values shown are FDR‐corrected for multiple comparisons; only *p*_FDR_ < 0.05 are marked with an asterisk. The numerical value in each cell represents the correlation coefficient (*r*) between the PVS VF of corresponding brain region and cognitive function. Color coding represents correlation direction and strength: blue (negative), red (positive); deeper colors indicate stronger correlations. The color bar on the right illustrates the correspondence between colors and correlation coefficients. BG, basal ganglia; BMI, body mass index; CSVD, cerebral small vessel disease; PVS, perivascular space; VF, volume fraction. *p < 0.05 after FDR correction.

## 4 Discussion

This study systematically investigated regional PVS alterations in CSVD patients and, for the first time, utilized BG subfield parcellation to investigate regional correlations with demographics, neuroimaging markers, and cognitive performance. Our findings reveal that CSVD patients exhibited significant PVS enlargement within the BG, particularly in the caudate and putamen subfields, and hippocampus. PVS volume in these regions was correlated with age and sex, with males demonstrating a greater PVS burden. Hippocampal PVS volume inversely correlated with hippocampal volume, and larger BG‐PVS volume, especially in the putamen, was linked to poorer global cognition and processing speed.

The glymphatic system, a brain‐wide perivascular network that facilitates cerebrospinal fluid‐interstitial fluid exchange and metabolic waste clearance, has been increasingly implicated in the pathophysiology of CSVD (Ang et al. 2024; Ma and Han 2026). Perivascular spaces constitute the anatomical infrastructure of this clearance pathway (Cao et al. 2025). Within this framework, radiologically detectable PVS enlargement may reflect impaired perivascular fluid transport, which could in turn compromise the microstructural integrity of fronto‐striatal circuits (Hein et al. 2025; Hong et al. 2024). The region‐specific expansion of PVS within the putamen and caudate warrants a multi‐level pathophysiological explanation. Anatomically, these striatal structures are supplied by long, small‐caliber lenticulostriate arteries branching off the high‐flow middle cerebral artery, rendering them vulnerable as a hemodynamic watershed region (Pantoni 2010). This configuration renders them exquisitely vulnerable to the damaging effects of pulsatile stress and hypoperfusion in CSVD (Wardlaw, Smith, Biessels, et al. 2013; Wardlaw, Smith, and Dichgans 2013). Histologically, these penetrating arterioles may possess less robust perivascular support, predisposing them to dilation and remodeling (Brown et al. 2018). Pathophysiologically, the striatum exhibits high metabolic activity and generates substantial metabolic waste, creating heavy dependence on efficient glymphatic clearance (Zlokovic 2011; Rasmussen et al. 2018; Iliff et al. 2012). We hypothesize that the convergence of hemodynamic stress, structural vulnerability, and high metabolic demand creates a locus of heightened susceptibility where global glymphatic dysfunction in CSVD is focally amplified, leading to the prominent PVS enlargement we observed (Iadecola 2017).

Given the established links between PVS and both physiological aging and CSVD, we systematically evaluated PVS associations with demographic and vascular biomarkers. We observed a progressive age‐dependent increase in PVS volume in the BG and hippocampus in CSVD group, consistent with prior reports (Zhu, Dufouil, et al. 2010; Zhu, Tzourio, et al. 2010; Doubal et al. 2010). Longitudinal evidence from a community‐dwelling elderly cohort has further substantiated this age‐related trajectory, demonstrating that BG‐PVS enlargement progresses over time and exhibits accelerated augmentation in individuals with vascular risk factors (Sugai et al. 2024). This temporal dynamic highlights the basal ganglia as a region particularly vulnerable to age‐ and vascular‐driven perivascular remodeling, aligning with our observation of pronounced PVS expansion in the putamen and caudate subfields. A notable finding was the significant main effect of sex on PVS volume, with males exhibiting larger PVS volumes across multiple brain regions. The interaction analyses suggest that these sex differences do not vary significantly as a function of the five vascular risk factors examined. This sex difference may arise from the differential effects of sex hormones on cerebrovascular tone, inflammation, and astrocytic function (Zuloaga et al. 2012; Gonzales et al. 2009). The association between hippocampal PVS volume and hippocampal volume further revealed a sex‐divergent pattern. Overall, larger hippocampal PVS volume correlated with smaller hippocampal volume, an effect driven predominantly by females. This female‐specific coupling may stem from postmenopausal estrogen loss, which compromises vasoprotective and neurotrophic support (Rettberg et al. 2014; Ramirez et al. 2016), thereby heightening vascular vulnerability to concurrent PVS enlargement and atrophy (Barisano et al. 2021; Evans et al. 2023). In males, by contrast, PVS enlargement appeared more closely tied to systemic vascular risk factors or androgen‐related pathways than to hippocampal volume loss, a dissociation consistent with the higher prevalence of hypertension and smoking in males (Kario et al. 2024). Although we adjusted for these factors, residual confounding cannot be fully excluded. Mechanistically, age‐related vascular stiffening may promote PVS enlargement through blood–brain barrier disruption and impaired clearance (Menze et al. 2024; Gertje et al. 2021), while concurrent hypoperfusion may contribute to hippocampal atrophy, which may be differentially modulated by sex. Notably, the absence of sex effects in the HC group is most parsimoniously explained by the limited sample size, which provided insufficient statistical power to detect sex differences of a magnitude comparable to those observed in the CSVD cohort. Accordingly, we positioned the HC group analyses as exploratory, emphasizing that this cohort was primarily intended to serve as a baseline reference for cognitive and imaging characteristics, and that its sample size precludes independent, robust inferences regarding sex‐related subgroup effects. Collectively, these findings underscore an intriguing sex‐divergent pathophysiology that warrants replication in larger cohorts with comprehensive hormonal profiling and longitudinal designs.

While prior work linked ePVS to hypertension, particularly in the BG (Potter et al. 2015), we did not replicate this association. Detailed assessment of medication use revealed substantial antihypertensive treatment rates (79.07%) with adequate control in the majority (64.71%). This therapeutic management may have attenuated the hypertensive burden on PVS, suggesting that effective blood pressure control can partly mitigate its pathological impact on the perivascular compartment. Our inability to replicate the hypertension‐PVS association may also reflect that local cerebral perfusion, rather than systemic blood pressure per se, is the more proximate determinant of BG‐PVS burden, as suggested by the independent association between BG‐PVS and reduced cerebral perfusion (Wang et al. 2024). Alternative nonhypertensive mechanisms, such as aging‐related glymphatic dysfunction, neuroinflammation, or vascular wall remodeling, may drive PVS enlargement in this established CSVD cohort. Future studies in broader populations incorporating longitudinal blood‐pressure trajectories and multimodal imaging biomarkers are warranted to clarify these relationships further.

At present, the mechanisms linking PVS enlargement to cognitive decline remain incompletely understood, but may involve glymphatic dysfunction as a central driver. As the anatomical substrate of the glymphatic system, radiologically evident PVS dilation may reflect impaired perivascular fluid transport, promoting neurotoxin accumulation and neuroinflammation (Rasmussen et al. 2018; Tian et al. 2023; Iliff et al. 2012; Nedergaard and Goldman 2020). Our study revealed region‐specific associations between PVS burden and deficits in distinct cognitive domains, interpretable through underlying neuroanatomical circuits. Specifically, the association between basal ganglia PVS, particularly in the dorsal striatum (putamen and caudate), and processing speed deficits aligns with the established role of these structures within cortico‐striato‐thalamo‐cortical (CSTC) loops, which are fundamental for executive function and information processing speed (Gunaydin and Kreitzer 2016; Bostan and Strick 2018; Parent and Hazrati 1995). PVS enlargement in this region may reflect local glymphatic dysfunction that disrupts the delicate neurochemical and electrophysiological balance required for rapid relay through these circuits (Nedergaard and Goldman 2020). This effect may be compounded by microangiopathic damage to adjacent frontostriatal white matter tracts, further impairing neural signaling and information gating, and ultimately manifesting as behavioral slowing (Wardlaw, Smith, Biessels, et al. 2013; Wardlaw, Smith, and Dichgans 2013; Duering et al. 2014).

Hippocampal PVS volume correlated inversely with visuospatial function, an association less commonly reported, yet potentially explicable through the role of the hippocampus within a broader spatial processing network. Specifically, the posterior hippocampal region contributes to spatial layout processing and maintains strong connections with parietal regions central to visuospatial integration (Uncapher and Wagner 2009). PVS enlargement within the hippocampal region may disrupt this network connectivity, thereby impairing integrated spatial processing (Raichle 2015). This network‐level interpretation is further supported by the multidomain nature of complex visuospatial tasks such as the clock drawing test, which rely not only on perceptual processing but also on spatial planning and memory, functions critically dependent on medial temporal lobe integrity (Burgess et al. 2002; Bird and Burgess 2008). Additionally, our hippocampal segmentation may have included adjacent parahippocampal cortex, a region essential for scene perception and contextual spatial processing (Epstein and Baker 2019). Collectively, these circuit‐ and network‐level interpretations provide a mechanistic framework for the regional specificity of our findings, positioning both basal ganglia and hippocampal PVS burden as potential biomarkers of vascular pathology affecting distinct cognitive domains. Notably, a composite scoring system incorporating MRI‐based glymphatic markers, including PVS burden, has demonstrated superior diagnostic performance in discriminating MCI from normal cognition in CSVD patients compared to individual markers alone (Xu et al. 2025), supporting the translational potential of PVS quantification as a clinically applicable component of multi‐marker diagnostic strategies.

The region‐specific associations between PVS burden and cognitive deficits support a circuit‐based framework in which glymphatic dysfunction may contribute to cognitive impairment. However, the biological interpretation of PVS enlargement should consider multiple mechanisms beyond glymphatic failure alone. First, hemodynamic stress from arterial pulsatility and stiffening may directly remodel the perivascular compartment. Second, neuroinflammation may contribute to perivascular tissue changes. Third, tissue atrophy and perivascular myelin loss may produce ex‐vacuo changes that mimic disease‐related PVS expansion. These processes may operate in parallel or sequentially during CSVD progression. Among these, structural brain changes warrant particular emphasis. WMHs and reactive gliosis may bidirectionally affect PVS morphology through remodeling and inflammation, while regional atrophy introduces ex‐vacuo dilation, as suggested by the inverse correlation between hippocampal PVS and hippocampal volume. Although our NABT‐based normalization mitigates measurement bias, it cannot eliminate indirect effects from adjacent lesional tissue. Nevertheless, the independent PVS‐cognition association persisting after volume adjustment supports its pathological significance beyond mere epiphenomenon. Collectively, these structural considerations underscore that PVS volume fraction reflects a convergence of pathogenic processes, necessitating longitudinal and multimodal approaches to disentangle causality.

Several limitations should be acknowledged. First, the single‐center design and modest sample size require validation in larger multicenter cohorts. Second, the cross‐sectional design prevents causal conclusions, necessitating longitudinal studies to determine directionality. Third, while our automated PVS quantification offers superior reproducibility, the Frangi filter approach has inherent limitations including parameter dependency and false‐positive susceptibility. Fourth, VRFs were analyzed as binary variables, which may limit the ability to detect dose–response relationships and cannot fully exclude residual confounding. Although we provided treatment status and control data for contextualization, future studies are warranted to employ continuous exposure metrics, such as cumulative blood pressure burden and longitudinal glycemic control indices, to more accurately delineate these associations. Fifth, hippocampal segmentation may have included the adjacent parahippocampal cortex, which participates in scene processing, potentially explaining the visuospatial association. Last, despite methodological controls for WMH and atrophy, PVS enlargement may reflect true pathology, ex‐vacuo dilatation or gliosis‐related remodeling. Future studies combining ultra‐high‐field MRI, dynamic contrast‐enhanced imaging, and histopathological validation are essential to clarify causality.

## 5 Conclusion

In conclusion, our study demonstrated elevated BG‐PVS volumes in CSVD patients, particularly within the putamen and caudate subfields, which were correlated with cognitive decline, positioning PVS as a promising therapeutic target to CSVD‐related cognitive impairment. Understanding PVS pathophysiology may illuminate glymphatic system dysfunction in CSVD pathogenesis, offering mechanistic insights to guide intervention strategies.

## Author Contributions

**Lulu Ai:** writing – original draft, methodology, investigation, software, formal analysis, data curation, conceptualization. **Zhiwei Li:** writing – review and editing, methodology, software, formal analysis, data curation, conceptualization. **Chaojuan Huang:** writing – review and editing, methodology, investigation, data curation, conceptualization. **Xia Zhou:** writing – review and editing, project administration, funding acquisition, formal analysis, data curation. **Qiaoqiao Xu:** writing – review and editing, project administration, funding acquisition, supervision, resources, data curation. **Xiaoqun Zhu:** writing – review and editing, project administration, funding acquisition, supervision, resources, data curation. **Zhongwu Sun:** writing – review and editing, project administration, funding acquisition, supervision, resources, data curation.

## Funding

This work was supported by the National Natural Science Foundation of China (82401420) and Natural Science Foundation of Anhui Province (2108085MH274), Anhui university scientific research major project (2022AH040159), Key Research and Development Projects of Anhui Province (202104j07020031), the Traditional Chinese Medicine Inheritance and Innovation Research Project of Anhui Province (2024CCCX230), and the Hefei City Health Commission applied medicine project (Hwk2023yb003).

## Supporting information


**Data S1:** Supporting Information eMethod.
**Table S1:** Comparison of regional PVS volume fraction between the HC and CSVD groups.
**Table S2:** Partial correlation analysis of PVS characteristics with clinical characteristics and neuroimaging markers in CSVD group.
**Table S3:** Interaction effects between sex and vascular risk factors (VRFs) on PVS volume fraction across four brain regions in CSVD group.
**Table S4:** Partial correlation analysis of PVS characteristics with age and sex in HC group.
**Table S5:** Partial correlation analysis of regional PVS volume fraction with cognition in CSVD group.

## Data Availability

The data that support the findings of this study are available from the corresponding author upon reasonable request.

## References

[hbm70642-bib-0001] Ang, P. S. , D. M. Zhang , S. A. Azizi , S. A. Norton de Matos , and J. R. Brorson . 2024. “The Glymphatic System and Cerebral Small Vessel Disease.” Journal of Stroke and Cerebrovascular Diseases 33, no. 3: 107557. 10.1016/j.jstrokecerebrovasdis.2024.107557. PMC11579894 38198946

[hbm70642-bib-0002] Ballerini, L. , R. Lovreglio , M. D. C. Valdés Hernández , et al. 2018. “Perivascular Spaces Segmentation in Brain MRI Using Optimal 3D Filtering.” Scientific Reports 8, no. 1: 2132. 10.1038/s41598-018-19781-5. PMC5794857 29391404

[hbm70642-bib-0003] Barisano, G. , N. Sheikh‐Bahaei , M. Law , A. W. Toga , and F. Sepehrband . 2021. “Body Mass Index, Time of Day and Genetics Affect Perivascular Spaces in the White Matter.” Journal of Cerebral Blood Flow and Metabolism 41, no. 7: 1563–1578. 10.1177/0271678x20972856. PMC8221772 33183133

[hbm70642-bib-0004] Benjamin, P. , S. Trippier , A. J. Lawrence , et al. 2018. “Lacunar Infarcts, but Not Perivascular Spaces, Are Predictors of Cognitive Decline in Cerebral Small‐Vessel Disease.” Stroke 49, no. 3: 586–593. 10.1161/strokeaha.117.017526. PMC5832012 29438074

[hbm70642-bib-0005] Benveniste, H. , X. Liu , S. Koundal , S. Sanggaard , H. Lee , and J. Wardlaw . 2019. “The Glymphatic System and Waste Clearance With Brain Aging: A Review.” Gerontology 65, no. 2: 106–119. 10.1159/000490349. PMC6329683 29996134

[hbm70642-bib-0006] Bird, C. M. , and N. Burgess . 2008. “The Hippocampus and Memory: Insights From Spatial Processing.” Nature Reviews. Neuroscience 9, no. 3: 182–194. 10.1038/nrn2335. 18270514

[hbm70642-bib-0007] Bostan, A. C. , and P. L. Strick . 2018. “The Basal Ganglia and the Cerebellum: Nodes in an Integrated Network.” Nature Reviews. Neuroscience 19, no. 6: 338–350. 10.1038/s41583-018-0002-7. PMC6503669 29643480

[hbm70642-bib-0008] Brown, R. , H. Benveniste , S. E. Black , et al. 2018. “Understanding the Role of the Perivascular Space in Cerebral Small Vessel Disease.” Cardiovascular Research 114, no. 11: 1462–1473. 10.1093/cvr/cvy113. PMC6455920 29726891

[hbm70642-bib-0009] Burgess, N. , E. A. Maguire , and J. O'Keefe . 2002. “The Human Hippocampus and Spatial and Episodic Memory.” Neuron 35, no. 4: 625–641. 10.1016/s0896-6273(02)00830-9. 12194864

[hbm70642-bib-0010] Cao, T. , C. Yang , J. Zhang , et al. 2025. “The Underlying Role of Pericyte‐Related Cerebral Lymphatic Microcirculation Dysfunction in Cerebral Small Vessel Disease.” Neurobiology of Disease 216: 107101. 10.1016/j.nbd.2025.107101. 40946810

[hbm70642-bib-0011] Debette, S. , S. Schilling , M. G. Duperron , et al. 2019. “Clinical Significance of Magnetic Resonance Imaging Markers of Vascular Brain Injury: A Systematic Review and Meta‐Analysis.” JAMA Neurology 76, no. 1: 81–94. 10.1001/jamaneurol.2018.3122. PMC6439887 30422209

[hbm70642-bib-0012] Ding, X. B. , X. X. Wang , D. H. Xia , et al. 2021. “Impaired Meningeal Lymphatic Drainage in Patients With Idiopathic Parkinson's Disease.” Nature Medicine 27, no. 3: 411–418. 10.1038/s41591-020-01198-1. 33462448

[hbm70642-bib-0013] Doubal, F. N. , A. M. MacLullich , K. J. Ferguson , et al. 2010. “Enlarged Perivascular Spaces on MRI Are a Feature of Cerebral Small Vessel Disease.” Stroke 41, no. 3: 450–454. 10.1161/strokeaha.109.564914. 20056930

[hbm70642-bib-0014] Dubost, F. , H. Adams , G. Bortsova , et al. 2019. “3D Regression Neural Network for the Quantification of Enlarged Perivascular Spaces in Brain MRI.” Medical Image Analysis 51: 89–100. 10.1016/j.media.2018.10.008. 30390514

[hbm70642-bib-0015] Duering, M. , G. J. Biessels , A. Brodtmann , et al. 2023. “Neuroimaging Standards for Research Into Small Vessel Disease—Advances Since 2013.” Lancet Neurology 22, no. 7: 602–618. 10.1016/s1474-4422(23)00131-x. 37236211

[hbm70642-bib-0016] Duering, M. , B. Gesierich , S. Seiler , et al. 2014. “Strategic White Matter Tracts for Processing Speed Deficits in Age‐Related Small Vessel Disease.” Neurology 82, no. 22: 1946–1950. 10.1212/wnl.0000000000000475. PMC4105258 24793184

[hbm70642-bib-0017] ElSayed, N. A. , G. Aleppo , V. R. Aroda , et al. 2023. “Classification and Diagnosis of Diabetes: Standards of Care in Diabetes—2023.” Diabetes Care 46, no. Suppl 1: S19–S40. 10.2337/dc23-S002. PMC9810477 36507649

[hbm70642-bib-0018] Epstein, R. A. , and C. I. Baker . 2019. “Scene Perception in the Human Brain.” Annual Review of Vision Science 5: 373–397. 10.1146/annurev-vision-091718-014809. PMC6989029 31226012

[hbm70642-bib-0019] Evans, T. E. , M. J. Knol , P. Schwingenschuh , et al. 2023. “Determinants of Perivascular Spaces in the General Population: A Pooled Cohort Analysis of Individual Participant Data.” Neurology 100, no. 2: e107–e122. 10.1212/wnl.0000000000201349. PMC9841448 36253103

[hbm70642-bib-0020] Gertje, E. C. , D. van Westen , C. Panizo , et al. 2021. “Association of Enlarged Perivascular Spaces and Measures of Small Vessel and Alzheimer Disease.” Neurology 96, no. 2: e193–e202. 10.1212/wnl.0000000000011046. 33046608

[hbm70642-bib-0021] Gonzales, R. J. , S. P. Duckles , and D. N. Krause . 2009. “Dihydrotestosterone Stimulates Cerebrovascular Inflammation Through NFκB, Modulating Contractile Function.” Journal of Cerebral Blood Flow and Metabolism 29, no. 2: 244–253. 10.1038/jcbfm.2008.115. PMC2658637 18941467

[hbm70642-bib-0022] Gottesman, R. F. , A. L. Schneider , Y. Zhou , et al. 2017. “Association Between Midlife Vascular Risk Factors and Estimated Brain Amyloid Deposition.” JAMA 317, no. 14: 1443–1450. 10.1001/jama.2017.3090. PMC5921896 28399252

[hbm70642-bib-0023] Greenberg, S. M. , and A. Charidimou . 2018. “Diagnosis of Cerebral Amyloid Angiopathy: Evolution of the Boston Criteria.” Stroke 49, no. 2: 491–497. 10.1161/strokeaha.117.016990. PMC5892842 29335334

[hbm70642-bib-0024] Grundy, S. M. , N. J. Stone , A. L. Bailey , et al. 2019. “2018 AHA/ACC/AACVPR/AAPA/ABC/ACPM/ADA/AGS/APhA/ASPC/NLA/PCNA Guideline on the Management of Blood Cholesterol: A Report of the American College of Cardiology/American Heart Association Task Force on Clinical Practice Guidelines.” Circulation 139, no. 25: e1082–e1143. 10.1161/cir.0000000000000625. PMC7403606 30586774

[hbm70642-bib-0025] Gunaydin, L. A. , and A. C. Kreitzer . 2016. “Cortico‐Basal Ganglia Circuit Function in Psychiatric Disease.” Annual Review of Physiology 78: 327–350. 10.1146/annurev-physiol-021115-105355. 26667072

[hbm70642-bib-0026] Hein, Z. M. , M. F. F. Arbain , M. D. C. Ramli , M. D. Che Ramli , U. Jaffer , and C. M. N. Che Mohd Nassir . 2025. “Glymphopathy and Reduced Processing Speed in Community‐Dwelling Adults With Silent Cerebral Small Vessel Disease: A DTI‐ALPS Study.” Journal of Clinical Medicine 14, no. 17: 6039. 10.3390/jcm14176039. PMC12429084 40943799

[hbm70642-bib-0027] Hong, H. , L. Hong , X. Luo , et al. 2024. “The Relationship Between Amyloid Pathology, Cerebral Small Vessel Disease, Glymphatic Dysfunction, and Cognition: A Study Based on Alzheimer's Disease Continuum Participants.” Alzheimer's Research & Therapy 16, no. 1: 43. 10.1186/s13195-024-01407-w. PMC10877805 38378607

[hbm70642-bib-0028] Hshieh, T. T. , W. F. Jung , L. J. Grande , et al. 2018. “Prevalence of Cognitive Impairment and Association With Survival Among Older Patients With Hematologic Cancers.” JAMA Oncology 4, no. 5: 686–693. 10.1001/jamaoncol.2017.5674. PMC5885202 29494732

[hbm70642-bib-0029] Huang, L. , K. L. Chen , B. Y. Lin , et al. 2018. “Chinese Version of Montreal Cognitive Assessment Basic for Discrimination Among Different Severities of Alzheimer's Disease.” Neuropsychiatric Disease and Treatment 14: 2133–2140. 10.2147/ndt.S174293. PMC6110265 30174426

[hbm70642-bib-0030] Huang, L. , X. Chen , W. Sun , et al. 2020. “Early Segmental White Matter Fascicle Microstructural Damage Predicts the Corresponding Cognitive Domain Impairment in Cerebral Small Vessel Disease Patients by Automated Fiber Quantification.” Frontiers in Aging Neuroscience 12: 598242. 10.3389/fnagi.2020.598242. PMC7829360 33505302

[hbm70642-bib-0031] Hurford, R. , A. Charidimou , Z. Fox , L. Cipolotti , R. Jager , and D. J. Werring . 2014. “MRI‐Visible Perivascular Spaces: Relationship to Cognition and Small Vessel Disease MRI Markers in Ischaemic Stroke and TIA.” Journal of Neurology, Neurosurgery, and Psychiatry 85, no. 5: 522–525. 10.1136/jnnp-2013-305815. PMC3995332 24249785

[hbm70642-bib-0032] Iadecola, C. 2017. “The Neurovascular Unit Coming of Age: A Journey Through Neurovascular Coupling in Health and Disease.” Neuron 96, no. 1: 17–42. 10.1016/j.neuron.2017.07.030. PMC5657612 28957666

[hbm70642-bib-0033] Iglesias, J. E. , J. C. Augustinack , K. Nguyen , et al. 2015. “A Computational Atlas of the Hippocampal Formation Using Ex Vivo, Ultra‐High Resolution MRI: Application to Adaptive Segmentation of In Vivo MRI.” NeuroImage 115: 117–137. 10.1016/j.neuroimage.2015.04.042. PMC4461537 25936807

[hbm70642-bib-0034] Iliff, J. J. , M. Wang , Y. Liao , et al. 2012. “A Paravascular Pathway Facilitates CSF Flow Through the Brain Parenchyma and the Clearance of Interstitial Solutes, Including Amyloid β.” Science Translational Medicine 4: 147ra111. 10.1126/scitranslmed.3003748. PMC3551275 22896675

[hbm70642-bib-0035] Javierre‐Petit, C. , M. Kontzialis , S. E. Leurgans , D. A. Bennett , J. A. Schneider , and K. Arfanakis . 2024. “Quantitative Assessment of Enlarged Perivascular Spaces via Deep‐Learning in Community‐Based Older Adults Reveals Independent Associations With Vascular Neuropathologies, Vascular Risk Factors and Cognition.” Brain Communications 6, no. 4: fcae252. 10.1093/braincomms/fcae252. PMC11316207 39130513

[hbm70642-bib-0036] Kario, K. , A. Okura , S. Hoshide , and M. Mogi . 2024. “The WHO Global Report 2023 on Hypertension Warning the Emerging Hypertension Burden in Globe and Its Treatment Strategy.” Hypertension Research 47, no. 5: 1099–1102. 10.1038/s41440-024-01622-w. 38443614

[hbm70642-bib-0037] Ma, Y. , and Y. Han . 2026. “Targeting the Brain's Glymphatic Pathway: A Novel Therapeutic Approach for Cerebral Small Vessel Disease.” Neural Regeneration Research 21, no. 2: 433–442. 10.4103/NRR.NRR-D-24-00821. PMC12220683 39688573

[hbm70642-bib-0038] Menze, I. , J. Bernal , P. Kaya , et al. 2024. “Perivascular Space Enlargement Accelerates in Ageing and Alzheimer's Disease Pathology: Evidence From a Three‐Year Longitudinal Multicentre Study.” Alzheimer's Research & Therapy 16, no. 1: 242. 10.1186/s13195-024-01603-8. PMC11526621 39482759

[hbm70642-bib-0039] Nedergaard, M. , and S. A. Goldman . 2020. “Glymphatic Failure as a Final Common Pathway to Dementia.” Science 370, no. 6512: 50–56. 10.1126/science.abb8739. PMC8186542 33004510

[hbm70642-bib-0040] Nishizawa, K. , J. Fujimori , and I. Nakashima . 2022. “Two‐Dimensional Measurements With Cut‐Off Values Are Useful for Assessing Brain Volume, Physical Disability, and Processing Speed in Multiple Sclerosis.” Multiple Sclerosis and Related Disorders 59: 103543. 10.1016/j.msard.2022.103543. 35078126

[hbm70642-bib-0041] Pantoni, L. 2010. “Cerebral Small Vessel Disease: From Pathogenesis and Clinical Characteristics to Therapeutic Challenges.” Lancet Neurology 9, no. 7: 689–701. 10.1016/s1474-4422(10)70104-6. 20610345

[hbm70642-bib-0042] Paradise, M. , J. D. Crawford , B. C. P. Lam , et al. 2021. “Association of Dilated Perivascular Spaces With Cognitive Decline and Incident Dementia.” Neurology 96, no. 11: e1501–e1511. 10.1212/wnl.0000000000011537. PMC8032377 33504642

[hbm70642-bib-0043] Parent, A. , and L. N. Hazrati . 1995. “Functional Anatomy of the Basal Ganglia. I. The Cortico‐Basal Ganglia‐Thalamo‐Cortical Loop.” Brain Research. Brain Research Reviews 20, no. 1: 91–127. 10.1016/0165-0173(94)00007-c. 7711769

[hbm70642-bib-0044] Passiak, B. S. , D. Liu , H. A. Kresge , et al. 2019. “Perivascular Spaces Contribute to Cognition Beyond Other Small Vessel Disease Markers.” Neurology 92, no. 12: e1309–e1321. 10.1212/wnl.0000000000007124. PMC6511092 30814324

[hbm70642-bib-0045] Patenaude, B. , S. M. Smith , D. N. Kennedy , and M. Jenkinson . 2011. “A Bayesian Model of Shape and Appearance for Subcortical Brain Segmentation.” NeuroImage 56, no. 3: 907–922. 10.1016/j.neuroimage.2011.02.046. PMC3417233 21352927

[hbm70642-bib-0046] Potter, G. M. , F. N. Doubal , C. A. Jackson , et al. 2015. “Enlarged Perivascular Spaces and Cerebral Small Vessel Disease.” International Journal of Stroke 10, no. 3: 376–381. 10.1111/ijs.12054. PMC4463944 23692610

[hbm70642-bib-0047] Putcha, D. , M. Brickhouse , D. A. Wolk , and B. C. Dickerson . 2019. “Fractionating the Rey Auditory Verbal Learning Test: Distinct Roles of Large‐Scale Cortical Networks in Prodromal Alzheimer's Disease.” Neuropsychologia 129: 83–92. 10.1016/j.neuropsychologia.2019.03.015. PMC6571167 30930301

[hbm70642-bib-0048] Raichle, M. E. 2015. “The Brain's Default Mode Network.” Annual Review of Neuroscience 38: 433–447. 10.1146/annurev-neuro-071013-014030. 25938726

[hbm70642-bib-0049] Ramirez, J. , C. Berezuk , A. A. McNeely , F. Gao , J. A. McLaurin , and S. E. Black . 2016. “Imaging the Perivascular Space as a Potential Biomarker of Neurovascular and Neurodegenerative Diseases.” Cellular and Molecular Neurobiology 36, no. 2: 289–299. 10.1007/s10571-016-0343-6. PMC11482437 26993511

[hbm70642-bib-0050] Ramirez, J. , C. Berezuk , A. A. McNeely , C. J. M. Scott , F. Gao , and S. E. Black . 2015. “Visible Virchow‐Robin Spaces on Magnetic Resonance Imaging of Alzheimer's Disease Patients and Normal Elderly From the Sunnybrook Dementia Study.” Journal of Alzheimer's Disease 43, no. 2: 415–424. 10.3233/jad-132528. 25096616

[hbm70642-bib-0051] Rasmussen, M. K. , H. Mestre , and M. Nedergaard . 2018. “The Glymphatic Pathway in Neurological Disorders.” Lancet Neurology 17, no. 11: 1016–1024. 10.1016/s1474-4422(18)30318-1. PMC6261373 30353860

[hbm70642-bib-0052] Rettberg, J. R. , J. Yao , and R. D. Brinton . 2014. “Estrogen: A Master Regulator of Bioenergetic Systems in the Brain and Body.” Frontiers in Neuroendocrinology 35, no. 1: 8–30. 10.1016/j.yfrne.2013.08.001. PMC4024050 23994581

[hbm70642-bib-0053] Scarpina, F. , and S. Tagini . 2017. “The Stroop Color and Word Test.” Frontiers in Psychology 8: 557. 10.3389/fpsyg.2017.00557. PMC5388755 28446889

[hbm70642-bib-0054] Sepehrband, F. , G. Barisano , N. Sheikh‐Bahaei , et al. 2019. “Image Processing Approaches to Enhance Perivascular Space Visibility and Quantification Using MRI.” Scientific Reports 9: 12351. 10.1038/s41598-019-48910-x. PMC6710285 31451792

[hbm70642-bib-0055] Sugai, Y. , T. Hiraka , A. Shibata , et al. 2024. “The Time‐Course Augmentation of Perivascular Space Enlargement in the Basal Ganglia Among a Community‐Dwelling Elder Population.” Japanese Journal of Radiology 42, no. 10: 1110–1121. 10.1007/s11604-024-01595-3. PMC11442546 38896331

[hbm70642-bib-0056] Ter Telgte, A. , E. M. C. van Leijsen , K. Wiegertjes , et al. 2018. “Cerebral Small Vessel Disease: From a Focal to a Global Perspective.” Nature Reviews. Neurology 14, no. 7: 387–398. 10.1038/s41582-018-0014-y. 29802354

[hbm70642-bib-0057] Tian, Y. , X. Cai , Y. Zhou , et al. 2023. “Impaired Glymphatic System as Evidenced by Low Diffusivity Along Perivascular Spaces Is Associated With Cerebral Small Vessel Disease: A Population‐Based Study.” Stroke and Vascular Neurology 8, no. 5: 413–423. 10.1136/svn-2022-002191. PMC10647865 37045543

[hbm70642-bib-0058] Uncapher, M. R. , and A. D. Wagner . 2009. “Posterior Parietal Cortex and Episodic Encoding: Insights From fMRI Subsequent Memory Effects and Dual‐Attention Theory.” Neurobiology of Learning and Memory 91, no. 2: 139–154. 10.1016/j.nlm.2008.10.011. PMC2814803 19028591

[hbm70642-bib-0059] van Norden, A. G. , K. F. de Laat , R. A. Gons , et al. 2011. “Causes and Consequences of Cerebral Small Vessel Disease. The RUN DMC Study: A Prospective Cohort Study. Study Rationale and Protocol.” BMC Neurology 11: 29. 10.1186/1471-2377-11-29. PMC3053228 21356112

[hbm70642-bib-0060] Wang, M. , G. Zhao , Y. Jiang , et al. 2023. “Disconnection of Network Hubs Underlying the Executive Function Deficit in Patients With Ischemic Leukoaraiosis.” Journal of Alzheimer's Disease 94, no. 4: 1577–1586. 10.3233/jad-230048. 37458032

[hbm70642-bib-0061] Wang, S. , S. Yang , D. Liang , et al. 2024. “Association Between Enlarged Perivascular Spaces in Basal Ganglia and Cerebral Perfusion in Elderly People.” Frontiers in Neurology 15: 1428867. 10.3389/fneur.2024.1428867. PMC11259966 39036638

[hbm70642-bib-0062] Wardlaw, J. M. , C. Smith , and M. Dichgans . 2013. “Mechanisms of Sporadic Cerebral Small Vessel Disease: Insights From Neuroimaging.” Lancet Neurology 12, no. 5: 483–497. 10.1016/s1474-4422(13)70060-7. PMC3836247 23602162

[hbm70642-bib-0063] Wardlaw, J. M. , C. Smith , and M. Dichgans . 2019. “Small Vessel Disease: Mechanisms and Clinical Implications.” Lancet Neurology 18, no. 7: 684–696. 10.1016/s1474-4422(19)30079-1. 31097385

[hbm70642-bib-0064] Wardlaw, J. M. , E. E. Smith , G. J. Biessels , et al. 2013. “Neuroimaging Standards for Research Into Small Vessel Disease and Its Contribution to Ageing and Neurodegeneration.” Lancet Neurology 12, no. 8: 822–838. 10.1016/s1474-4422(13)70124-8. PMC3714437 23867200

[hbm70642-bib-0065] Waymont, J. M. J. , M. D. C. Valdés Hernández , J. Bernal , et al. 2024. “Systematic Review and Meta‐Analysis of Automated Methods for Quantifying Enlarged Perivascular Spaces in the Brain.” NeuroImage 297: 120685. 10.1016/j.neuroimage.2024.120685. 38914212

[hbm70642-bib-0066] Williams, B. , G. Mancia , W. Spiering , et al. 2018. “2018 ESC/ESH Guidelines for the Management of Arterial Hypertension.” European Heart Journal 39, no. 33: 3021–3104. 10.1093/eurheartj/ehy339. 30165516

[hbm70642-bib-0067] Williams, B. W. , W. Mack , and V. W. Henderson . 1989. “Boston Naming Test in Alzheimer's Disease.” Neuropsychologia 27, no. 8: 1073–1079. 10.1016/0028-3932(89)90186-3. 2797414

[hbm70642-bib-0068] Xu, J. , Y. Su , J. Fu , Y. Shen , Q. Dong , and X. Cheng . 2023. “Glymphatic Pathway in Sporadic Cerebral Small Vessel Diseases: From Bench to Bedside.” Ageing Research Reviews 86: 101885. 10.1016/j.arr.2023.101885. 36801378

[hbm70642-bib-0069] Xu, M. , B. Zhang , H. Mao , et al. 2025. “MRI‐Based Glymphatic Markers and Composite Scoring for the Discrimination of Mild Cognitive Impairment in Cerebral Small Vessel Disease.” Neuroradiology 67, no. 12: 3599–3612 10.1007/s00234‐025‐03836‐2. 10.1007/s00234-025-03836-2 41204954

[hbm70642-bib-0070] Yamamoto, E. A. , J. H. Bagley , M. Geltzeiler , et al. 2024. “The Perivascular Space Is a Conduit for Cerebrospinal Fluid Flow in Humans: A Proof‐Of‐Principle Report.” Proceedings of the National Academy of Sciences of the United States of America 121, no. 42: e2407246121. 10.1073/pnas.2407246121. PMC11494350 39374384

[hbm70642-bib-0071] Yao, M. , Y. C. Zhu , A. Soumaré , et al. 2014. “Hippocampal Perivascular Spaces Are Related to Aging and Blood Pressure but Not to Cognition.” Neurobiology of Aging 35, no. 9: 2118–2125. 10.1016/j.neurobiolaging.2014.03.021. 24731517

[hbm70642-bib-0072] Zhang, Y. , M. Brady , and S. Smith . 2001. “Segmentation of Brain MR Images Through a Hidden Markov Random Field Model and the Expectation‐Maximization Algorithm.” IEEE Transactions on Medical Imaging 20, no. 1: 45–57. 10.1109/42.906424. 11293691

[hbm70642-bib-0073] Zhu, Y. C. , C. Dufouil , A. Soumaré , B. Mazoyer , H. Chabriat , and C. Tzourio . 2010. “High Degree of Dilated Virchow‐Robin Spaces on MRI Is Associated With Increased Risk of Dementia.” Journal of Alzheimer's Disease 22, no. 2: 663–672. 10.3233/jad-2010-100378. 20847444

[hbm70642-bib-0074] Zhu, Y. C. , C. Tzourio , A. Soumaré , B. Mazoyer , C. Dufouil , and H. Chabriat . 2010. “Severity of Dilated Virchow‐Robin Spaces Is Associated With Age, Blood Pressure, and MRI Markers of Small Vessel Disease: A Population‐Based Study.” Stroke 41, no. 11: 2483–2490. 10.1161/strokeaha.110.591586. 20864661

[hbm70642-bib-0075] Zlokovic, B. V. 2011. “Neurovascular Pathways to Neurodegeneration in Alzheimer's Disease and Other Disorders.” Nature Reviews. Neuroscience 12, no. 12: 723–738. 10.1038/nrn3114. PMC4036520 22048062

[hbm70642-bib-0076] Zuloaga, K. L. , D. T. O'Connor , R. J. Handa , et al. 2012. “Estrogen Receptor Beta Dependent Attenuation of Cytokine‐Induced Cyclooxygenase‐2 by Androgens in Human Brain Vascular Smooth Muscle Cells and Rat Mesenteric Arteries.” Steroids 77, no. 8–9: 835–844. 10.1016/j.steroids.2012.04.013. PMC3809122 22542504

