# Supplementary material for: Association of Perivascular Space Volume in Basal Ganglia Subfields With Cognitive Impairment in Cerebral Small Vessel Disease
Source: Hum Brain Mapp. 2026 Nov 9;47(16):e70642. doi: 10.1002/hbm.70642 (PMC13746381; doi:10.1002/hbm.70642)
Supplement: Supplementary file 1 [file HBM-47-e70642-s001.docx]

**Supplementary Information**

**Association of perivascular space volume in basal ganglia subfields with cognitive impairment in cerebral small vessel disease**

Lulu Ai^1, †^, Zhiwei Li^1, †^, Chaojuan Huang^1^, Xia Zhou^1^, Qiaoqiao Xu^2, *^, Xiaoqun Zhu^1, *^, Zhongwu Sun^1, *^

^1^Department of Neurology, The First Affiliated Hospital of Anhui Medical University, Hefei, China.

^2^Department of Neurology, The Third Affiliated Hospital of Anhui Medical University (Hefei City First People’s Hospital), Hefei, China.

†These authors have contributed equally to this work.

*Corresponding author:

**CONTACT INFO**: Zhongwu Sun and Xiaoqun Zhu, Department of Neurology, The First Affiliated Hospital of Anhui Medical University, 218 Jixi Road, Hefei, Anhui 230022, China. Tel.: +86 0551 62922328.

Qiaoqiao Xu, Department of Neurology, The Third Affiliated Hospital of Anhui Medical University (Hefei City First People’s Hospital), 390 Huaihe Road, Hefei, Anhui 230071, China. Tel.: +86 0551 62186777.

E-mails: sunzhwu@126.com (Zhongwu Sun), zxq_ayfy@163.com (Xiaoqun Zhu), [xqq7039@126.com](mailto:xqq7039@126.com) (Qiaoqiao Xu).

**Supplementary eMethod**

**MRI Data Acquisition**

MRI imaging data were obtained using a 3.0-Tesla MR system (Discovery MR750w, General Electric, Milwaukee, WI, USA) equipped with a 24-channel head coil. To reduce scanning noise, participants wore earplugs and foam padding restricted head movement. Simultaneously, participants were instructed to remain awake with eyes closed in a relaxed state. High resolution three-dimensional T1-weighted (3D-T1) images were acquired through a brain volume (BRAVO) sequence with the following parameters: slice thickness = 1.0 mm, repetition time (TR) = 8.464 ms, echo time (TE) = 3.248 ms, inversion time (TI) = 450 ms, flip angle (FA) = 12°, field of view (FOV) = 256 mm × 256 mm, matrix size = 256 × 256, slice thickness = 1 mm without gap, 188 sagittal slices and acquisition time = 296 s. T2 fluid-attenuated inversion recovery (T2 FLAIR) images were acquired with the following parameters: TR = 9000 ms, TE = 119.84 ms, FA = 160◦, FOV = 225 mm×225 mm, matrix size = 512×512, number of layers = 19, layer thickness = 7 mm and acquisition time = 1 min 57 s.

**Supplementary Tables**

Table S1 Comparison of regional PVS volume fraction between the HC and CSVD groups.

|  | HC  (n=40) | CSVD  (n=132) | T/λ-value | *P* value |
| --- | --- | --- | --- | --- |
| BG-PVS VF (%) | 0.063 ± 0.009 | 0.073 ± 0.010 | -5.065 | **<0.001** |
| Amygdala-PVS VF | 0.004 ± 0.001 | 0.005 ± 0.001 | -1.406 | 0.162 |
| Caudate-PVS VF | 0.019 ± 0.004 | 0.022 ± 0.005 | -3.459 | **<0.001** |
| Pallidum-PVS VF | 0.011 ± 0.004 | 0.010 ± 0.003 | 0.071 | 0.944 |
| Putamen-PVS VF | 0.022 ± 0.008 | 0.028 ± 0.007 | -3.670 | **<0.001** |
| Thalamus-PVS VF (%) | 0.066 ± 0.008 | 0.070 ± 0.010 | -1.219 | 0.225 |
| HP-PVS VF (%) | 0.011 ± 0.004 | 0.014 ± 0.005 | -2.379 | **0.019** |
| WM-PVS VF (%) | 1.752 ± 0.334 | 1.834 ± 0.632 | -0.693 | 0.489 |

Note: Results are expressed as means ± standard deviation. Group comparisons were performed adjusting for age and sex. *P* values shown are unadjusted for multiple comparisons, given the exploratory nature of this analysis. Bold values indicate *P* < 0.05 (uncorrected).

Abbreviations: HC, healthy control; CSVD, cerebral small vessel disease; PVS, perivascular spaces; VF, volume fraction; BG, basal ganglia; HP, hippocampal; WM, white matter; WMH, white matter hyperintensity.

PVS volume fraction = PVS volume / (grey+ white matter volume-WMH volume) *100%

Table S2 Partial correlation analysis of PVS characteristics with clinical characteristics and neuroimaging markers in CSVD group.

|  | BG-PVS VF | | |  | HP-PVS VF | | |  | Thalamus-PVS VF | | |  | WM-PVS VF | | |
| --- | --- | --- | --- | --- | --- | --- | --- | --- | --- | --- | --- | --- | --- | --- | --- |
|  | r | *P* | *P*_FDR_ |  | r | *P* | *P*_FDR_ |  | r | *P* | *P*_FDR_ |  | r | *P* | *P*_FDR_ |
| Demographic characteristics | | | | | | | | | | | | | | | |
| age | 0.24 | 0.006 | **0.044** |  | 0.33 | <0.001 | **0.002** |  | 0.20 | 0.020 | 0.124 |  | 0.03 | 0.436 | 0.735 |
| sex | 0.33 | <0.001 | **0.002** |  | 0.38 | <0.001 | **<0.001** |  | 0.24 | 0.006 | **0.044** |  | 0.01 | 0.904 | 0.925 |
| Vascular risk factors | | | | | | | | | | | | | | | |
| Hypertension | 0.04 | 0.653 | 0.809 |  | -0.01 | 0.958 | 0.958 |  | 0.10 | 0.276 | 0.675 |  | 0.04 | 0.643 | 0.809 |
| Diabetes | 0.07 | 0.451 | 0.735 |  | 0.05 | 0.604 | 0.809 |  | 0.08 | 0.397 | 0.735 |  | -0.01 | 0.895 | 0.925 |
| hypercholesterolemia | 0.04 | 0.625 | 0.809 |  | -0.06 | 0.493 | 0.774 |  | 0.05 | 0.592 | 0.809 |  | -0.03 | 0.742 | 0.878 |
| Smoking | 0.11 | 0.221 | 0.659 |  | -0.05 | 0.585 | 0.809 |  | 0.08 | 0.352 | 0.735 |  | -0.01 | 0.891 | 0.925 |
| BMI | 0.18 | 0.036 | 0.197 |  | -0.07 | 0.412 | 0.735 |  | 0.01 | 0.883 | 0.925 |  | 0.17 | 0.048 | 0.221 |
| Neuroimaging markers | | | | | | | | | | | | | | | |
| WMH Fazekas scores | 0.11 | 0.230 | 0.659 |  | 0.07 | 0.431 | 0.735 |  | -0.11 | 0.240 | 0.659 |  | 0.04 | 0.662 | 0.809 |
| Lacunes | 0.07 | 0.415 | 0.735 |  | 0.08 | 0.352 | 0.735 |  | 0.10 | 0.255 | 0.659 |  | 0.11 | 0.233 | 0.659 |
| CMBs | -0.18 | 0.050 | 0.221 |  | 0.03 | 0.773 | 0.878 |  | -0.06 | 0.525 | 0.796 |  | 0.17 | 0.062 | 0.248 |
| HP volume | -0.08 | 0.352 | 0.735 |  | -0.26 | 0.004 | **0.043** |  | 0.03 | 0.778 | 0.878 |  | -0.11 | 0.224 | 0.659 |

Note: Partial correlation analyses were conducted to control for potential confounders. Statistical significance of correlation coefficients (r) was assessed, with multiple comparisons controlled using the false discovery rate (FDR) method. *P* values are uncorrected for multiple comparisons; *P*_FDR_ values are FDR-corrected. Bold values indicate *P*_FDR_ < 0.05. Abbreviations: CSVD, cerebral small vessel disease; PVS, perivascular spaces; VF, volume fraction; BG, basal ganglia; HP, hippocampal; WM, white matter; BMI, body mass index; WMH, white matter hyperintensity; CMBs, cerebral microbleeds.

Table S3. Interaction effects between sex and vascular risk factors (VRFs) on PVS volume fraction across four brain regions in CSVD group.

|  | VRFs | B (SE) | β | t | P | P_FDR_ | Partial η² | ΔR² |
| --- | --- | --- | --- | --- | --- | --- | --- | --- |
| BG-PVS VF (%) | Hypertension | 0.002 (0.003) | 0.100 | 0.715 | 0.476 | 0.774 | 0.004 | 0.002 |
|  | Diabetes | -0.001 (0.004) | -0.042 | -0.320 | 0.750 | 0.852 | 0.001 | <0.001 |
|  | hypercholesterolemia | -0.001 (0.003) | -0.032 | -0.330 | 0.742 | 0.852 | 0.001 | <0.001 |
|  | Smoking | 0.005 (0.006) | 0.258 | 0.920 | 0.359 | 0.774 | 0.007 | 0.004 |
|  | BMI | 0.001 (0.001) | 0.263 | 2.751 | **0.007** | 0.136 | 0.057 | 0.033 |
| Thalamus-PVS VF (%) | Hypertension | 0.001 (0.003) | 0.028 | 0.187 | 0.852 | 0.852 | <0.001 | <0.001 |
|  | Diabetes | -0.003 (0.005) | -0.094 | -0.689 | 0.492 | 0.774 | 0.004 | 0.003 |
|  | hypercholesterolemia | 0.001 (0.004) | 0.024 | 0.235 | 0.815 | 0.852 | <0.001 | <0.001 |
|  | Smoking | 0.014 (0.007) | 0.612 | 2.085 | **0.039** | 0.391 | 0.033 | 0.022 |
|  | BMI | <0.001 (0.001) | 0.059 | 0.560 | 0.576 | 0.823 | 0.003 | 0.002 |
| HP-PVS VF (%) | Hypertension | 0.001 (0.001) | 0.111 | 0.802 | 0.424 | 0.774 | 0.005 | 0.003 |
|  | Diabetes | 0.001 (0.002) | 0.091 | 0.707 | 0.481 | 0.774 | 0.004 | 0.002 |
|  | hypercholesterolemia | -0.002 (0.002) | -0.091 | -0.958 | 0.340 | 0.774 | 0.007 | 0.004 |
|  | Smoking | -0.001 (0.003) | -0.141 | -0.501 | 0.618 | 0.823 | 0.002 | 0.001 |
|  | BMI | <0.001 (0.000) | 0.100 | 1.024 | 0.308 | 0.774 | 0.008 | 0.005 |
| WM-PVS VF (%) | Hypertension | -0.343 (0.205) | -0.261 | -1.675 | 0.096 | 0.584 | 0.022 | 0.017 |
|  | Diabetes | -0.066 (0.294) | -0.033 | -0.224 | 0.823 | 0.852 | <0.001 | <0.001 |
|  | hypercholesterolemia | 0.166 (0.247) | 0.073 | 0.672 | 0.503 | 0.774 | 0.004 | 0.003 |
|  | Smoking | -0.675 (0.427) | -0.502 | -1.579 | 0.117 | 0.584 | 0.019 | 0.015 |
|  | BMI | -0.049 (0.035) | -0.151 | -1.369 | 0.173 | 0.693 | 0.015 | 0.011 |

Note: All models were adjusted for age and total intracranial volume. B = unstandardized regression coefficient; SE = standard error; β = standardized coefficient; ΔR² = R² increment after introducing the interaction term. P values were adjusted using Benjamini–Hochberg false discovery rate (FDR-BH) method.

Abbreviations: VRFs, vascular risk factors; CSVD, cerebral small vessel disease; PVS, perivascular spaces; VF, volume fraction; BG, basal ganglia; HP, hippocampal; WM, white matter.

Table S4 Partial correlation analysis of PVS characteristics with age and sex in HC group.

|  | Subgroup | BG-PVS VF | | |  | HP-PVS VF | | |  | Thalamus-PVS VF | | |  | WM-PVS VF | | |
| --- | --- | --- | --- | --- | --- | --- | --- | --- | --- | --- | --- | --- | --- | --- | --- | --- |
|  |  | r | *P* | *P*_FDR_ |  | r | *P* | *P*_FDR_ |  | r | *P* | *P*_FDR_ |  | r | *P* | *P*_FDR_ |
| Age | All participants | 0.47 | 0.002 | **0.035** |  | 0.40 | 0.010 | 0.081 |  | 0.31 | 0.050 | 0.160 |  | -0.18 | 0.255 | 0.341 |
|  | Male participants | 0.39 | 0.104 | 0.237 |  | 0.47 | 0.041 | 0.160 |  | 0.28 | 0.238 | 0.341 |  | -0.30 | 0.208 | 0.332 |
|  | Female  participants | 0.50 | 0.020 | 0.108 |  | 0.30 | 0.184 | 0.327 |  | 0.30 | 0.181 | 0.327 |  | -0.17 | 0.458 | 0.564 |
| sex | All participants | 0.12 | 0.499 | 0.570 |  | 0.32 | 0.063 | 0.168 |  | 0.00 | 0.994 | 0.994 |  | -0.02 | 0.934 | 0.994 |

Note: Partial correlation analyses were conducted to control for potential confounders. The statistical significance of correlation coefficients (r) was assessed, and *P*-values were corrected for multiple comparisons using the false discovery rate (FDR) method. Bold values indicate *P* < 0.05.

Abbreviations: HC, healthy control; PVS, perivascular spaces; VF, volume fraction; BG, basal ganglia; HP, hippocampal; WM, white matter.

Table S5 Partial correlation analysis of regional PVS volume fraction with cognition in CSVD group

|  | Global cognitive function | | |  | Memory function | | |  | Executive function | | |  | Processing speed | |  |  | Language function | |  |  | Visuospatial function | |  | |  |
| --- | --- | --- | --- | --- | --- | --- | --- | --- | --- | --- | --- | --- | --- | --- | --- | --- | --- | --- | --- | --- | --- | --- | --- | --- | --- |
|  | r | *P* | *P*_FDR_ |  | r | *P* | *P*_FDR_ |  | r | *P* | *P*_FDR_ |  | r | *P* | *P*_FDR_ |  | r | *P* | *P*_FDR_ |  | r | *P* | *P*_FDR_ | |  |
| Model 1 | | | | | | | | | | | | | | | | | | | | | | | |  |  |
| BG | -0.26 | 0.004 | **0.037** |  | -0.07 | 0.443 | 0.610 |  | -0.20 | 0.030 | 0.130 |  | -0.31 | <0.001 | **0.007** |  | -0.06 | 0.535 | 0.681 |  | -0.23 | 0.011 | 0.064 | |  |
| Amygdala | -0.09 | 0.321 | 0.562 |  | -0.05 | 0.622 | 0.768 |  | -0.06 | 0.525 | 0.681 |  | -0.04 | 0.664 | 0.795 |  | 0.002 | 0.980 | 0.995 |  | -0.19 | 0.034 | 0.130 | |  |
| Caudate | -0.01 | 0.916 | 0.961 |  | -0.02 | 0.820 | 0.907 |  | -0.15 | 0.103 | 0.277 |  | -0.25 | 0.005 | **0.037** |  | -0.10 | 0.270 | 0.539 |  | 0.02 | 0.807 | 0.907 | |  |
| Pallidum | 0.001 | 0.95 | 0.995 |  | 0.16 | 0.082 | 0.245 |  | 0.09 | 0.308 | 0.562 |  | 0.04 | 0.681 | 0.795 |  | 0.15 | 0.106 | 0.277 |  | -0.08 | 0.397 | 0.610 | |  |
| Putamen | -0.32 | <0.001 | **0.007** |  | -0.07 | 0.450 | 0.610 |  | -0.17 | 0.061 | 0.202 |  | -0.19 | 0.034 | 0.130 |  | -0.07 | 0.422 | 0.610 |  | -0.26 | 0.005 | **0.037** | |  |
| HP | -0.20 | 0.027 | 0.130 |  | -0.10 | 0.284 | 0.542 |  | -0.17 | 0.063 | 0.202 |  | -0.14 | 0.123 | 0.303 |  | -0.11 | 0.221 | 0.492 |  | -0.35 | <0.001 | **0.003** | |  |
| Thalamus | 0.07 | 0.444 | 0.610 |  | 0.11 | 0.223 | 0.492 |  | -0.08 | 0.405 | 0.610 |  | -0.10 | 0.262 | 0.539 |  | 0.09 | 0.340 | 0.571 |  | -0.01 | 0.909 | 0.961 | |  |
| Model 2 | | | | | | | | | | | | | | | | | | | | | | | |  |  |
| BG | -0.27 | 0.003 | **0.027** |  | -0.05 | 0.557 | 0.680 |  | -0.20 | 0.034 | 0.143 |  | -0.31 | <0.001 | **0.010** |  | -0.06 | 0.550 | 0.680 |  | -0.23 | 0.011 | 0.068 | |  |
| Amygdala | -0.07 | 0.445 | 0.649 |  | -0.05 | 0.623 | 0.726 |  | -0.09 | 0.336 | 0.609 |  | -0.07 | 0.464 | 0.649 |  | 0.03 | 0.773 | 0.867 |  | -0.15 | 0.095 | 0.284 | |  |
| Caudate | -0.07 | 0.461 | 0.649 |  | 0.001 | 0.989 | 0.989 |  | -0.14 | 0.121 | 0.298 |  | -0.24 | 0.009 | 0.064 |  | -0.12 | 0.186 | 0.435 |  | 0.01 | 0.950 | 0.973 | |  |
| Pallidum | -0.01 | 0.872 | 0.939 |  | 0.15 | 0.117 | 0.298 |  | 0.06 | 0.506 | 0.680 |  | 0.01 | 0.920 | 0.966 |  | 0.14 | 0.121 | 0.298 |  | -0.09 | 0.339 | 0.609 | |  |
| Putamen | -0.31 | <0.001 | **0.010** |  | -0.07 | 0.435 | 0.649 |  | -0.19 | 0.041 | 0.158 |  | -0.22 | 0.016 | 0.086 |  | -0.06 | 0.538 | 0.680 |  | -0.24 | 0.009 | 0.064 | |  |
| HP | -0.16 | 0.076 | 0.245 |  | -0.09 | 0.348 | 0.609 |  | -0.20 | 0.033 | 0.143 |  | -0.17 | 0.061 | 0.212 |  | -0.09 | 0.321 | 0.609 |  | -0.33 | <0.001 | **0.010** | |  |
| Thalamus | 0.05 | 0.566 | 0.680 |  | 0.10 | 0.281 | 0.609 |  | -0.08 | 0.392 | 0.649 |  | -0.10 | 0.302 | 0.609 |  | 0.07 | 0.427 | 0.649 |  | -0.03 | 0.784 | 0.867 | |  |

Note: Partial correlation analyses were conducted to control for potential confounders. Model 1 was adjusted for demographic (age, sex, and years of education), VRFs (hypertension, diabetes, hypercholesterolemia, smoking, and BMI) and CSVD neuroimaging markers (WMHs, lacunes, and CMBs). Model 2 was additionally adjusted for respective brain region volumes. Statistical significance of correlation coefficients (r) was assessed, with multiple comparisons controlled using the false discovery rate (FDR) method. P values are uncorrected for multiple comparisons; *P*_FDR_ values are FDR-corrected. Bold values indicate *P*_FDR_ < 0.05. Abbreviations: PVS, perivascular spaces; CSVD, cerebral small vessel disease; BG, basal ganglia; HP, hippocampus.
